# Gas Bubbles from Biodegradable Magnesium Implants Convey Mechanical Cues and Promote Immune Cell Stimulation

**DOI:** 10.1002/advs.202503123

**Published:** 2025-05-11

**Authors:** Heithem Ben Amara, Jincy Philip, Omar Omar, Peter Thomsen

**Affiliations:** ^1^ Department of Biomaterials Institute of Clinical Sciences Sahlgrenska Academy University of Gothenburg Gothenburg SE‐405 30 Sweden; ^2^ Department of Biomedical Dental Sciences College of Dentistry Imam Abdulrahman bin Faisal University P.O. Box 1982 Dammam 31441 Saudi Arabia

**Keywords:** biodegradable implants, cavity, cellular mechanotransduction, gene expression profiling, immunohistochemistry, Immunomodulation, magnesium

## Abstract

In ever‐increasing numbers, patients are treated with biodegradable magnesium implants. While gas bubbles frequently arise in soft tissue overlying magnesium implants, their biological implications remain uncertain. This study investigates how bubble accumulation and evolution across various biological lengths and time scales influence adjacent tissue and cell behavior in rats. Bubbles accumulate in tissues around magnesium during initial postimplantation days, then fully resorb. Alterations in tissue and cell geometry around bubbles coincide with accumulation of cells, many with macrophage phenotypes, and increased expression of the mechanosensitive ion‐channel Piezo1. Using spatially resolved transcriptomics, strong proinflammatory pathway activation is revealed near bubbles with marked expression of the proliferative macrophage marker secreted phosphoprotein 1 (*Spp1*). Spatial transcriptomics also reveals strong enrichment of cytoskeletal rearrangement genes, demonstrating that cells respond to mechanical cues from bubbles. Notably, both time and bubble–implant distance strongly influence the cellular response. Over time, as bubbles are located farther from the implant, regenerative processes decline, and inflammation predominates. These findings suggest that bubbles from magnesium implant degradation create an intricate local response influencing tissue healing through inflammatory and mechanical pathways. This study underscores the need for magnesium implants with controlled gas release and meticulous monitoring of bubble evolution in patients.

## Graphical abstract

Bubbles in soft tissue are the unavoidable consequence of magnesium implant degradation in patients, yet their biological significance remains unclear. Using advanced imaging and spatial transcriptomics, this study reveals a pronounced inflammatory and mechanical response near bubbles, contrasting with pro‐regenerative events closer to the implant. This spatial interplay underscores the need for controlled gas release and vigilant monitoring in patients.

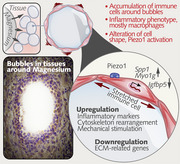

## 1 Introduction

Metallic implants made of magnesium are increasingly used in patients, serving as endovascular stents to restore the geometry of diseased vessels and as osteosynthesis systems to secure the repair of fractured bones. Owing to their in situ biodegradation ability and favorable mechanical properties, magnesium implants meet the need to overcome the flaws of their conventional permanent analogs: unwanted renarrowing of the stented vessel and inevitable surgical removal of the orthopedic implant after bone healing. To date, robust evidence has suggested that these implants can facilitate regeneration^[^ ^1^, ^2^, ^3^, ^4^ ^]^ via immunomodulation^[^ ^3^, ^5^, ^6^ ^]^ and achieve clinical success.^[^ ^7^, ^8^ ^]^

When it is implanted in the body, magnesium starts degrading immediately, releasing hydrogen gas into the surrounding tissues. This degradation results in a peculiar yet consistent morphological feature in the tissues around magnesium implants,^[^ ^9^ ^]^ i.e., the accumulation of radiolucent bubbles in the tissue environment during the initial course of implant degradation.^[^ ^10^, ^11^, ^12^ ^]^ In most instances where magnesium implants successfully integrate into the accommodating bone and soft tissue, these bubbles disappear with time. However, when implants fail, bubbles consistently accompany persistent inflammation of the soft tissues.^[^ ^13^, ^14^ ^]^ Therefore, questions are increasingly being raised about the effects of these bubbles on tissue healing.

Although bubble accumulation in soft tissues around magnesium implants has been reported since their initial use over a century ago,^[^ ^15^ ^]^ understanding how these bubbles influence the surrounding tissue and cellular response has remained a challenge.^[^ ^16^ ^]^

One prevailing belief is that these bubbles preclude healing around magnesium implants.^[^ ^17^ ^]^ Based on this assumption, some researchers have suggested the removal of bubbles from soft tissues via puncture in clinical^[^ ^18^ ^]^ and preclinical settings^[^ ^19^, ^20^ ^]^ to prevent complications caused by these bubbles. This viewpoint has also driven the design of magnesium‐based biomaterials with increased sophistication in controlling hydrogen release to prevent bubble generation.^[^ ^17^ ^]^

However, a contrasting standpoint questions the purported deleterious effects of bubbles, based on growing evidence demonstrating the multifaceted benefits of hydrogen. In fact, hydrogen‐based therapies have been shown to alleviate inflammation through the downregulation of key proinflammatory cytokines.^[^ ^21^ ^]^ In addition, hydrogen therapy was demonstrated to exert antiapoptotic and proangiogenic effects through mechanisms involving the downregulation of proapoptotic markers^[^ ^21^ ^]^ and the promotion of vascular endothelial growth factor^[^ ^22^ ^]^ expression. Nonetheless, it remains unclear whether bubbles in soft tissues interfacing with magnesium implants indeed contain hydrogen^[^ ^23^, ^24^ ^]^ adding to the ambiguity regarding their biological effects.

Addressing these questions becomes even more urgent considering the implications of bubbles in critical applications, such as stented vascular segments, or the debated use of magnesium implants in vulnerable patients.^[^ ^25^ ^]^ Therefore, our study investigates how the accumulation and evolution of these bubbles across various biological lengths and time scales influence the behavior of immediately adjacent tissues and cells. To achieve this goal, we employed spatially resolved modalities, including spatial transcriptomics, for detailed studies of cells and their molecular circuits in response to bubbles generated by magnesium implants in the nearby soft tissue of rats. By comparing cells in tissue territories near bubbles with those at the interface with the implant or in distant control regions across different time points, we demonstrate that bubbles modify the geometry of nearby tissues and cells, amplify inflammation, and trigger pathways related to mechanical stimulation. Furthermore, spatial transcriptomics revealed that this cellular response is largely influenced by the time after implantation and by the distance between the bubbles and the implant. Over time, as the bubbles are located far from the implant, regenerative processes diminish, and inflammation predominates around the bubbles, necessitating meticulous clinical monitoring of bubble evolution to optimize healing outcomes in patients.

## 2 Results

### 2.1 Bubble‐Like Cavities Accumulate in the Tissues Surrounding Magnesium Implants, and their Morphology Changes Over Time

**Figure 1:**
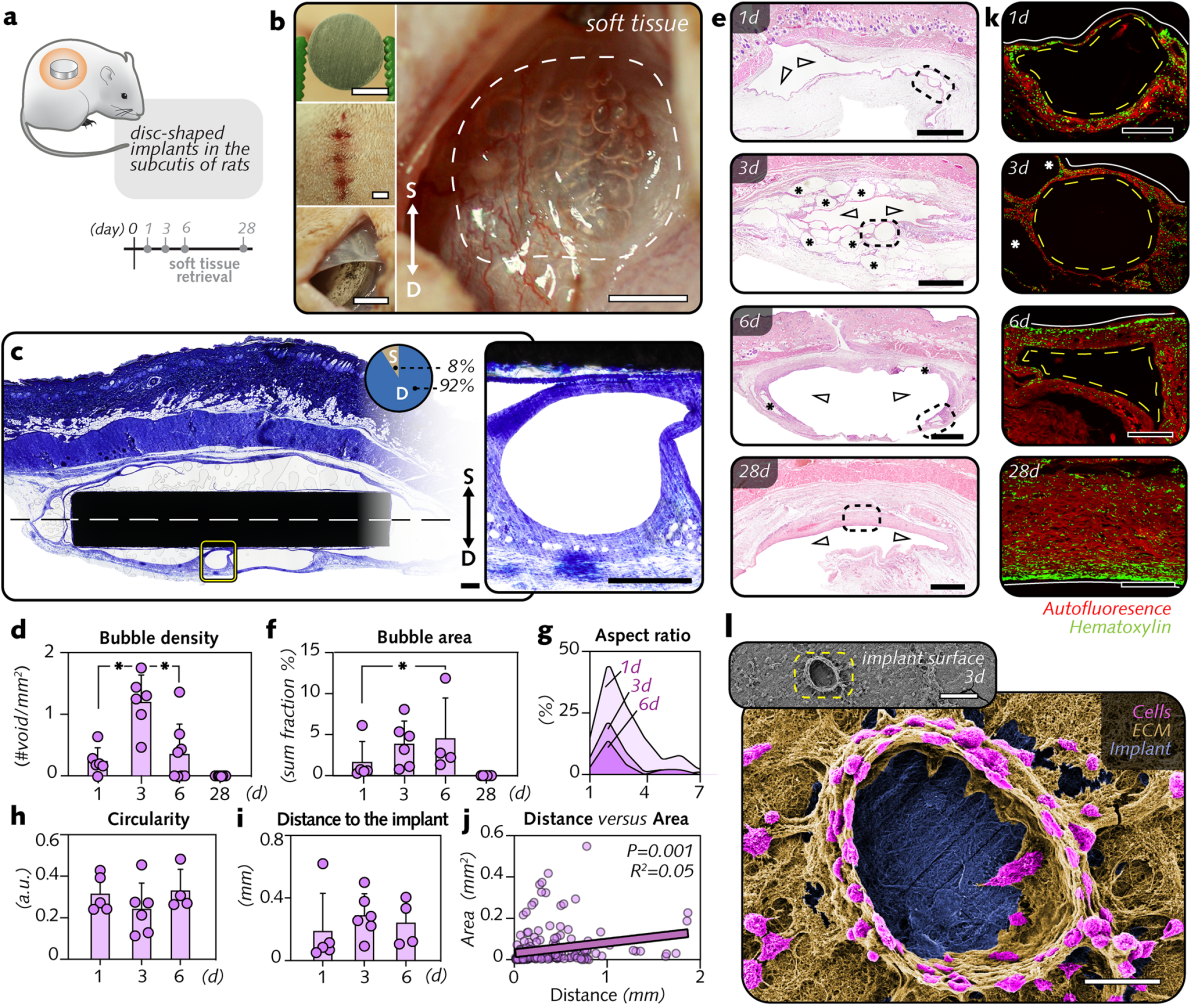
Bubbles accumulate quickly in soft tissue adjacent to magnesium implants and feature time‐related changes in their morphology. a) Disc‐shaped magnesium implants were inserted into the soft tissue of rats to monitor the 1–28 d‐peri‐implant tissue response. b) Photographs showing the subcutaneous implantation of a magnesium disc collected after 3 d, with several bubble‐shaped features (broken lines) discernible in the thickness of the peri‐implant fascia upon implant retrieval (*S = surface side; D = deep side*). c) Histological analysis by toluidine blue‐staining enabled the examination of peri‐implant bubbles, revealing their preferential arrangement in the subcutaneous fascia toward the deep side of the implant. The implant axis (broken white line) was used to demarcate the deep (*D*), and surface (*S*) sides of the implants. Bubbles were more frequent on the deep side (92%) in comparison with the surface side (8%). d) The density of bubbles measured by counting bubbles detected in soft tissue sections. The number of bubbles was divided by the total area of the subcutaneous fascia in soft tissue sections used for histomorphometry (n = 6/group/time point). e) Histological analysis of soft tissue at 1, 3, and 6 d (hematoxylin and eosin staining). f–h) Area (f, fraction of the summed bubble area relative to the total area of the subcutaneous fascia), aspect ratio (g), and circularity (h) of the bubbles (n = 5–6 per group per time point). i,j) Distance between the bubble and the implant interface and its association with the bubble area (simple linear regression; n = 5–6 per group per time point). k) Magnified autofluorescence images of the bubbles outlined with broken yellow lines in e. Arrowheads indicate the implant sites. The asterisks indicate bubbles in the peri‐implant soft tissue. The white lines indicate the interface with the implants. Hematoxylin‐stained nuclei are pseudocolorized in green. l) Pseudocolorized scanning electron microscopy image of the surface of a magnesium implant retrieved at 3 d postimplantation (ECM: Extracellular matrix). The data are shown as the means with error bars indicating the SD. *p < 0.05 between time points. Kruskal‒Wallis or unpaired Mann‒Whitney *U* test was used. Scale bars: b = 5 mm; c = 0.5 mm; e = 1 mm; k = 500 µm; l = 20 µm.

To examine the soft tissue response to bubbles formed during magnesium degradation, discs made of high‐purity magnesium (Figure S1, Supporting Information) were implanted subcutaneously in the dorsal skin of rats (**Figure** **1**a). Upon reentry of the surgical site after 1, 3, 6, and 28 d, macroscopic analysis of the tissues surrounding the magnesium implants in the initial postoperative days revealed numerous spheroid‐shaped features in the immediate peri‐implant tissue (Figure 1b; Figure S2, Supporting Information).

Reminiscent of bubbles generated by the gaseous products of magnesium implant degradation,^[^ ^26^ ^]^ these spheroids were detected in the subcutaneous fascia neighboring the magnesium implants. Histological analysis of the implants and associated tissue sections confirmed these macroscopic observations (Figure 1c). Specifically, cavities with a gross ellipsoid contour that expanded from the implants in soft tissue were observed; these cavities appeared in varying numbers and at varying distances. Histological examination also revealed that the location of the bubbles tended to be toward the deep side of the disc implants in soft tissues (Figure 1c). Their density was the highest at 3 d and then markedly declined (Figure 1d; Figure S3, Supporting Information). By 28 d, no bubbles were detected in the peri‐implant soft tissues.

To determine whether the changes in the density of the peri‐implant bubbles over time were accompanied by alterations in their morphology, we performed hematoxylin and eosin staining of sections of the peri‐implant soft tissues at 1, 3, 6, and 28 d (Figure 1e) for further morphological characterization. The total area occupied by the bubbles (Figure 1f; Figure S4, Supporting Information) increased ≈3‐fold from 1 to 6 d. However, no differences in the bubble perimeter over time were observed (Figure S4, Supporting Information). The stability of the aspect ratio (Figure 1g; Figure S4, Supporting Information) and circularity (Figure 1h; Figure S4, Supporting Information), averaging 2.5–3.8 and 2.4–3.3 from 1 to 6 d, respectively, revealed that the contours of the bubbles remained ellipsoidal without alteration over time. Similarly, the average distance between the bubble and the implant–soft tissue interface was comparable across time points, ranging from 0.187–0.288 mm (Figure 1i). A positive association existed between the area of the bubble and its distance to the implant–soft tissue interface (Figure 1j).

Notably, histological analysis (Figure 1k) revealed that the tissues closest to the bubbles displayed a typical adaptive organization of cells and the extracellular matrix. From 1 to 6 d, infiltration into the tissues surrounding the bubble was evident. This peribubble aggregation of cells was comparable to that in the tissues contacting the implants but contrasted with the scattered cells populating the peripheral loose connective tissue. A similar arrangement of cells and associated extracellular matrix around the bubbles was also observed via electron microscopy examination of the surface of the magnesium implants that were collected during the initial postimplantation days (Figure 1l).

Together, these observations indicate that bubble‐like cavities that formed due to the degradation of magnesium implants accumulate in the surrounding tissues, with significant morphological changes over time.

### 2.2 Inflammatory and Mechanotransduction Pathways are Activated in Cells Surrounding Bubbles

**Figure 2:**
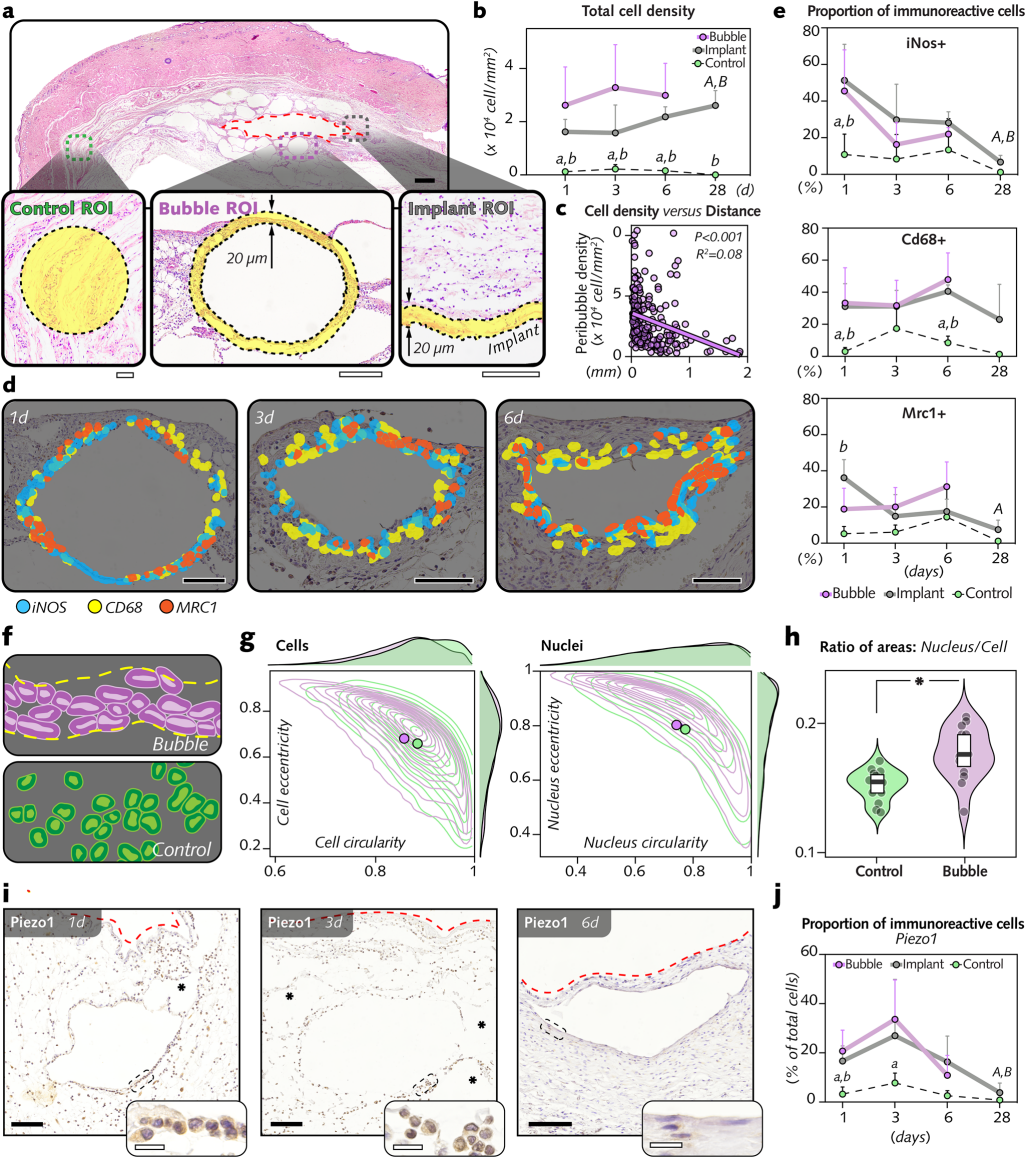
Bubbles in soft tissue promoted initial cellular infiltration, transiently increased inflammatory proteins, and elicited mechanosensitive Piezo1 immunoreactivity. a) Histological sections (hematoxylin and eosin) enabled the investigation of morphological and phenotypical changes caused by bubbles on surrounding cells. Yellow shading and broken lines mark the region of interest (ROI) for automated cell counting within 20 µm from each bubble. Comparisons were made with ROIs within 20 µm from the implant–tissue interface (*Implant*) and >1 mm from the interface (*Control;* circular region ≈45000 µm^2^, equal to average bubble area). b) Cell density in *Bubble*, *Implant*, and *Control* (n = 5–6 per group per time point). c) Associations between peribubble cell density and the bubble distance from the implant–tissue interface (linear regression; n = 5–6 per time point). d) Immunohistochemical analysis of iNos, Cd68, and Mrc1 in peribubble tissues. Images show cells immunoreactive to these markers (Figure S6, Supporting Information), pseudocolorized, and merged from consecutive sections. e) Percentages of immunopositive cells (n = 5–6 per group per time point). (f) QuPath segmentation highlights differences in cell shape near Bubble (yellow lines show Bubble ROI) versus Control. g) Contour plots comparing circularity/eccentricity of cells near bubbles (n = 201470 cells; purple) versus control (n = 1177 cells; green); average values noted in each plot. h) Ratio of average cell area to total nucleus area (n = 15; all time points pooled). i) Piezo1 immunostaining near bubbles. Red broken lines indicate implant–tissue interface. Asterisks indicate adjacent bubbles. j) Percentage of cells immunopositive for Piezo1 relative to total cells in bubbles and control ROIs (n = 5 per group per time point). The data are shown as the means with the error bars indicating the SD. * *p <* 0.05, bubble versus control. a) *p<*0.05, bubble versus control. b) *p <* 0.05, implant versus control. A) *p <* 0.05, 1 d versus 28 d in implant group. B) *p <* 0.05 3 d versus 28 d in Implant group. Friedman's two‐way ANOVA by rank test for paired comparisons of Bubble, Implant, and Control. Kruskal‒Wallis test for comparisons between time points. Scale bars: a: *black* = 1 mm, *white =* 50 µm; d = 50 µm; i: *black* = 50 µm, *white =* 20 µm.

To determine whether bubbles influence the phenotype of associated cells, histomorphometry was performed to study the cellularity in the areas nearest the bubbles surrounding the implants in soft tissues. For this purpose, cells within 20 µm of the bubble outline (**Figure** **2**a) were detected and counted. This total was subsequently compared to the number of cells within 20 µm from the implant–soft tissue interface and that in a distant, control region (Figure 2a).

The peribubble region featured the highest density of cells (3255 cells mm^−2^, Figure 2b; Figure S5, Supporting Information), surpassing that at the implant interface (1576 cells mm^−2^) and that in the distant control region (224 cells mm^−2^) by more than 2‐fold and 10‐fold, respectively, at 3 d. The density of cells populating the peribubble tissues was negatively associated with distance (Figure 2c).

Given the initial increase in the number of cells, we aimed to determine the phenotypes of the cells recruited to the peribubble region (Figure 2d,e). The immunoreactivity of cells in tissues within 20 µm of the bubbles demonstrated the expression of cluster of differentiation 68 (Cd68), an antigen specific to cells of the monocytic lineage, including circulating and tissue‐resident macrophages; inducible nitric oxide synthase (iNos), an intracellular inflammatory marker expressed by, among other cell types, proinflammatory macrophages; and mannose receptor C‐type 1 (Mrc1), a receptor that plays an important role in the alternative activation of macrophages (Figure 2d; Figure S6, Supporting Information). The percentages of iNos‐, Cd68‐, and Mrc1‐immunoreactive cells in the peribubble tissues (Figure 2e; Figure S6, Supporting Information) were noticeably greater than those in the control regions and similar to those in the tissues interfacing the implant. Together, the results suggested that the peribubble tissue initially exhibited greater cell density than the tissues at the implant interface. In addition, the peribubble tissue contained accumulated macrophages with different phenotypes, similar to those of cells recruited to the implant–soft tissue interface.

To observe the shape changes in the cells around the bubbles (Figure 2f), we compared the morphological characteristics of the cells in the bubble and control regions. Cells and their nuclei exhibited differences in their contours (Figure 2g; Figure S7, Supporting Information). Additionally, the ratio of the nucleus‐to‐cell area, which provides a measure of the relative nuclear size within the cell, was greater in the bubble group than in the control group and suggested a diminished cell area in the cells around the bubbles (Figure 2h). This finding implied that bubbles elicit changes in the shape of the surrounding cells, eventually through the mechanical stimuli that they exert. Upon mechanical activation, Piezo1 functions as an ion channel that allows passage to cations, and engages downstream pathways.^[^ ^27^ ^]^ Immunohistochemistry was used to determine the protein expression of Piezo1 by cells associated with bubbles. Higher proportions of Piezo1+ cells were detected near the bubbles and close to the implant–soft tissue interface than in the control regions at 1 and 3 d (Figure 2i,j, Figure S8, Supporting Information). Subsequently (i.e., at 6 and 28 d), fewer immunoreactive cells were detected around the bubbles and at the interface.

Together, these findings demonstrated that, in a comparable manner to implants, bubbles elicit the expression of inflammatory and mechanosensitive proteins.

### 2.3 Spatial Transcriptomics Reveals that Inflammation‐ and Cytoskeleton‐Related Pathways are the Main Transcriptional Programs Activated by Bubbles

**Figure 3:**
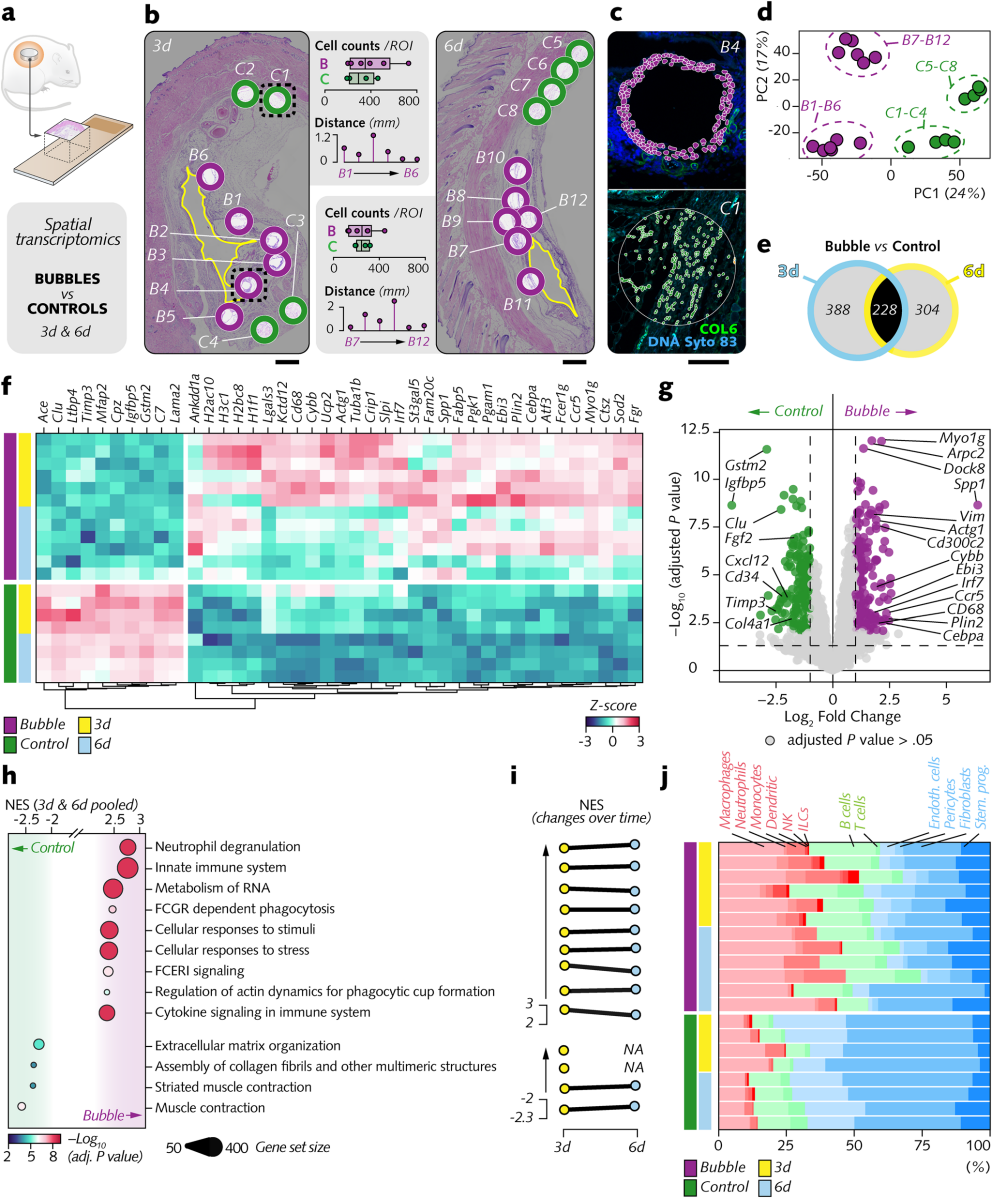
Transcriptomic profiling of cellular responses in the tissue surrounding magnesium implants in the peribubble and control regions. a) Spatial transcriptomics studied transcriptomic regulation by bubbles on nearby cells compared to control regions. b) Representative histological images of tissue sections showing regions of interest (ROIs) selected for spatial transcriptomics analysis around bubbles at 3 and 6 d. ROIs include peribubble regions (B1–B12) and distant control regions (C1–C8). Cell counts per ROI and distance measurements from the implant interface are annotated. ROIs were outlined via the GeoMx DSP instrument, yielding ≈14 million sequencing reads per ROI, processed and normalized via NanoString's analysis pipeline. c) Fluorescence images showing morphology marking with the NanoString GeoMx platform via the nuclear stain Syto 83 (blue) and immunostaining for COL6 (collagen type VI, green; extracellular matrix) in the peribubble and control ROIs. d) Principal component analysis (PCA) of gene expression data from the peribubble and control regions at 3 and 6 d. e) Venn diagram of differentially expressed genes (DEGs) between bubble and control regions, identifying 388 DEGs at 3 d and 304 DEGs at 6 d, with 228 shared across both time points. Differential expression analysis (DEA) was performed via linear mixed models. f) Heatmap showing DEGs shared across 3 and 6 d, organized by hierarchical clustering, with the highest coefficient of variance (CV) in the peribubble and control regions. g) Volcano plots depicting fold changes and significance of DEGs in control (left) and peribubble (right) regions. h) Pathway enrichment analysis of pooled DEGs (3 and 6 d) in peribubble regions via the clusterProfiler and ReactomePA packages. Node size indicates gene set size and color intensity reflects adjusted *p* values (NES = normalized enrichment score). i) Temporal dynamics of enriched pathways (NES = normalized enrichment score). j) Cell type deconvolution analysis via SpatialDecon package (dendritic = dendritic cells; NK cells = natural killer cells; ILCs = innate lymphoid cells; Endoth = endothelial cells; Stem prog = stem and progenitor cells). *p* < 0.05 was adjusted via the Benjamini‒Hochberg procedure. Scale bar: b = 1 mm; c = 50 µm.

To verify the regulatory effect of the bubble indicated by immunohistochemistry on nearby cells, we generated transcriptome‐wide spatial profiles in the peribubble and control regions in soft tissues surrounding magnesium implants in rats via NanoString GeoMx Digital Spatial Profiler (DSP) (**Figure** **3**a). We selected the 3 and 6 d time points to capture the initial inflammatory response and the early reparative phase following implantation.

Cells within 20 µm from six bubbles (*B*) at different distances from the implant–soft tissue interface and cells in four distant, control regions (*C*) were analyzed with spatially resolved transcriptomics in each tissue section (Figure 3b) upon morphology marker staining (DNA Syto 83: nuclei; COL6: extracellular matrix) (Figure 3c). The cell counts, averaging ≈300 cells per region of interest (ROI), were comparable between both regions. Approximately 14 million sequencing reads per ROI were generated on average. After quality control and normalization, principal component analysis (PCA) (Figure 3d) demonstrated evident region‐ and time‐point‐dependent clustering, indicating that the gene expression pattern in the cells of the peribubble region was different from that in the cells in the control region. Differential expression analysis (DEA) (Figure 3e) between the bubble and control regions revealed 388 and 304 differentially expressed genes (DEGs), respectively, with 228 DEGs conserved across both time points.

Unsupervised hierarchical clustering of these common DEGs (Figure 3f) revealed two distinct groups of genes with the highest coefficient of variation (CV), reflecting the highest expression dispersion across the profiled regions: *i)* genes downregulated by cells of the peribubble region, many of which are related to the immune response (*C7*, *Ltbp4*) and extracellular matrix remodeling (*Mfap2*, *Timp3*), and *ii)* genes upregulated in these cells, which are associated primarily with immune activation (*Cd68, Fcer1* *g*) and cytoskeleton organization (*Tubab1b*, *Actg1*). Many of these genes exhibited very strong differential expression between cells in the peribubble region and those in the control region (Figure 3g; Figure S9, Supporting Information). Notably, *Spp1—*a gene characteristic of proliferative macrophages involved in wound healing^[^ ^28^ ^]^ —and *Myo1g*—a key regulatory gene of immune cell elasticity through actin network deformation^[^ ^29^ ^]^—featured the greatest upregulation in the peribubble cells. Other highly upregulated genes included *Arpc2* and *Dock8*, which are both critical in the regulation of the shape of macrophages and T cells^[^ ^30^, ^31^ ^]^ respectively, as well as the immune response‐related gene markers *Irf7, Cybb*, and *Ccr5*. In contrast, the genes with the greatest downregulation included the fibroblast core signature gene linked to the production of the fibrotic extracellular matrix *Igfbp5* ^[^ ^32^, ^33^ ^]^ and the antioxidation gene marker *Gstm2*. Other strongly downregulated genes included the extracellular matrix‐related genes *Fgf2* and *Col4a1* as well as the gene markers of stem cell mobilization *Cxcl12* and *Cd34*.

The DEGs were further studied through pathway enrichment analysis via ReactomePA and clusterProfiler (Figure 3h). On the one hand, the elevated upregulation of pathways linked to the immune response (e.g., neutrophil degranulation, innate immune system, and FCGR‐related phagocytosis) and to the cellular response (cellular response to stimuli and cellular response to stress), and on the other hand, the downregulation of extracellular matrix‐related pathways (assembly of collagen fibrils and other multimeric structures, extracellular matrix organization, and muscle contraction) confirmed the dichotomic transcriptomic regulation in cells near the bubbles shown by the DEA. Most of the recruited pathways were conserved across time points (Figure 3i).

Furthermore, cell type deconvolution (Figure 3j; Figure S10, Supporting Information) revealed pronounced differences between the peribubble and control regions. In confirmation of the proinflammatory microenvironment of the bubbles, immune cells—particularly macrophages and B cells—represented the highest cell proportion around the bubbles. In contrast, fibroblasts were the predominant cell type in the control region.

Overall, spatial transcriptomics analysis confirmed that the cells surrounding the bubbles activate specific transcriptional programs predominantly related to inflammation and cytoskeleton organization.

### 2.4 Transcriptional Changes Induced by the Bubbles in Surrounding Cells Vary Depending on their Distance from the Implant

**Figure 4:**
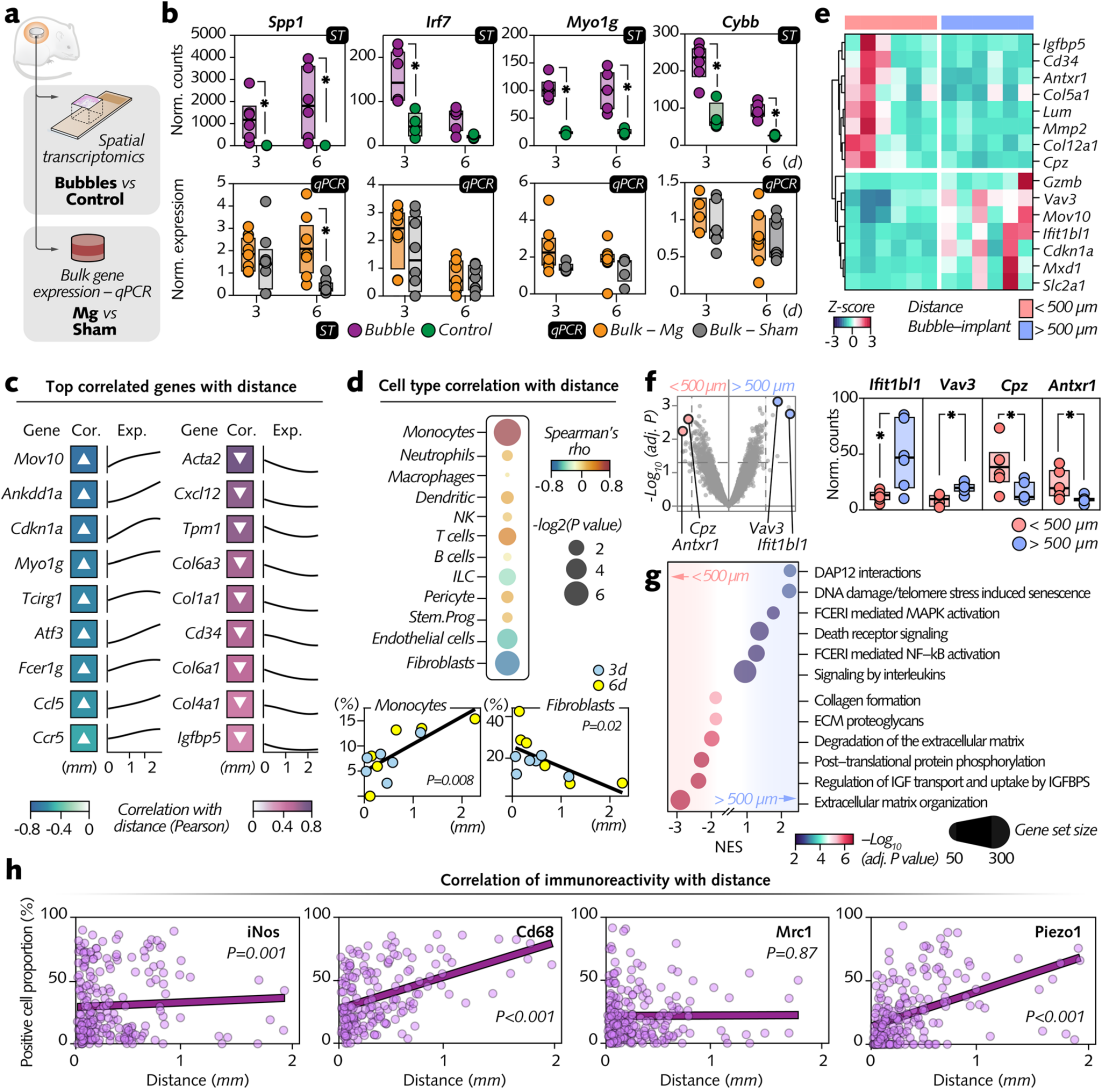
Distance‐dependent transcriptional and cellular responses in tissue surrounding magnesium implant bubbles. a) Bulk soft tissue punches were sampled at the interface with the magnesium implant (Mg) to perform quantitative PCR (qPCR) on genes highly upregulated by the bubbles, as indicated by spatial transcriptomics (ST). Comparisons were made with qPCR on tissue samples from sham‐wounded sites without implants. b) Comparison of gene expression in the peribubble region (ST) and bulk tissue (qPCR) surrounding the implants. c) Expression of differentially expressed genes (DEGs) in peribubble regions as a function of bubble distance from the implant. d) Relationships between cell types and distance in peribubble regions at 3 and 6 d, demonstrating a significant distance‐dependent distribution for monocytes and fibroblasts (Spearman correlation). e) High coefficient of variation (CV) genes in peribubble regions, grouped by proximity to the implant (<500 µm vs >500 µm; n = 6 in each group with 3 and 6 d pooled). f) Differential expression analysis (DEA) using linear mixed models of peribubble genes in the <500 µm and >500 µm groups. g) Pathway enrichment analysis comparing the >500 µm and <500 µm groups in the peribubble region via the *clusterProfiler* and *ReactomePA* packages. The node size indicates the gene set size, and the color intensity reflects adjusted *p* values (NES = normalized enrichment score). h) Correlation analysis (Pearson) between bubble distance from the implant and protein expression levels of iNos, Cd68, Mrc1, and Piezo1 confirming distance‐dependent transcriptional and protein responses. The data are presented as the means with the error bars indicating the SD. *p < 0.05, < 500 µm versus > 500 µm using unpaired Mann‒Whitney *U* test. *p* < 0.05 was adjusted via the Benjamini‒Hochberg procedure.

Given the strong upregulation of genes linked to the immune response and cytoskeleton organization in cells neighboring the bubbles, we questioned whether this upregulation was localized to the immediate vicinity of the bubbles or extended to the broader tissue upon magnesium implantation. To address this, we performed quantitative PCR (qPCR) analysis on bulk tissues surrounding magnesium implants and compared them to nonimplanted sham tissues (**Figure** **4**a; Figure S11, Supporting Information). We selected genes strongly upregulated by the bubbles: *Irf7* and *Cybb*, which are markers of the pro‐inflammatory response; *Spp1*, which is characteristic of proliferative macrophages; and *Myo1* *g*, which is a marker of alterations in the cellular shape. In contrast to spatial transcriptomics findings in peribubble cells—which exhibited up to an ≈2000‐fold increase in gene expression compared with that in control regions—none of these genes showed consistent differential expression in the bulk tissues around magnesium implants compared with that in the sham tissues (Figure 4b). This finding indicates that the upregulation of these genes is confined to cells immediately adjacent to the bubbles and does not occur throughout the surrounding tissue.

Recognizing the importance of spatial context in cellular responses to bubbles, we next investigated whether the distance of the bubbles from the implant influenced the expression levels of DEGs in neighboring cells (Figure 4c). We observed an increase in the expression of genes linked to the immune response (*Ccl5*, *Ccr5*, and *Fcer1* *g*) with increasing distance of the bubble from the implant. Conversely, genes related to matrix generation and remodeling (*Col1a1, Col6a3, Col4a1, Igfbp5, Cd34*, and *Cxcl12*) presented decreasing expression with increasing distance. The cell elasticity marker *Myo1* *g* was positively correlated with distance, whereas the other cytoskeleton‐related genes *Acta2* and *Tpm1* were negatively correlated.

We further assessed the proportion of specific cell types in relation to the distance of the bubbles from the implant (Figure 4d). At both 3 and 6 d, monocytes and fibroblasts were strongly correlated with distance but in opposite directions. Specifically, the proportion of monocytes around the bubbles increased as their distance from the implant increased, whereas the proportion of fibroblasts decreased under the same conditions.

These findings elucidated another important factor influencing transcriptomic regulation in cells near the bubbles: their distance from the implant. To further investigate this spatial influence, we selected a threshold distance of 500 µm from the implant to equally distribute bubbles at both 3 and 6 d into two groups: those close to the implant (< 500 µm; n = 6) and those farther away (> 500 µm; n = 6), establishing statistically relevant sample groups for comparative analysis. Notably, many genes with the highest CVs in the < 500 µm group, including *Igfbp5, Cd34, Col5a1, Col12a1, Mmp2, Lum, Antxr1*, and *Cpz*, were indicative of matrix assembly and remodeling. In contrast, many high‐CV genes in the > 500 µm group, such as *Gzmb* and *Ifit1bl1*, are markers of the immune response (Figure 4e). DEA further revealed that the DEGs with the strongest upregulation in the > 500 µm group were the cytoskeleton regulator *Vav3* and the interferon response gene *Ifit1bl1*. Conversely, in the < 500 µm group, the DEGs with the greatest upregulation were the matrix regulatory gene *Cpz* and the matrix mechanical sensor *Antxr1* (Figure 4f). Moreover, pathway analysis confirmed the activation of immune‐related pathways (e.g., FCERI‐mediated MAPK activation, FCERI‐mediated NF‐kB activation, and signaling by interleukins) in the > 500 µm group, whereas matrix‐related pathways (e.g., extracellular matrix organization, regulation of IGF transport and uptake by IGFBPs, degradation of the extracellular matrix, and collagen formation) were strongly activated in the < 500 µm group (Figure 4g).

Immunohistochemistry analysis further supported these findings, revealing a strong positive correlation between the distance of the bubbles from the implant and the percentage of cells immunopositive for iNos, Cd68, and Piezo1 (Figure 4h). This provided additional protein‐level evidence in support of the spatial transcriptomics data that distance influences cellular responses in the peribubble region.

Collectively, these results demonstrate that both the transcriptional and protein‐level changes induced by the bubbles are influenced by their distance from the implant.

### 2.5 Spatial and Temporal Segregation of Immune Activation and Matrix Organization in Peribubble Regions Versus the Implant Interface

**Figure 5:**
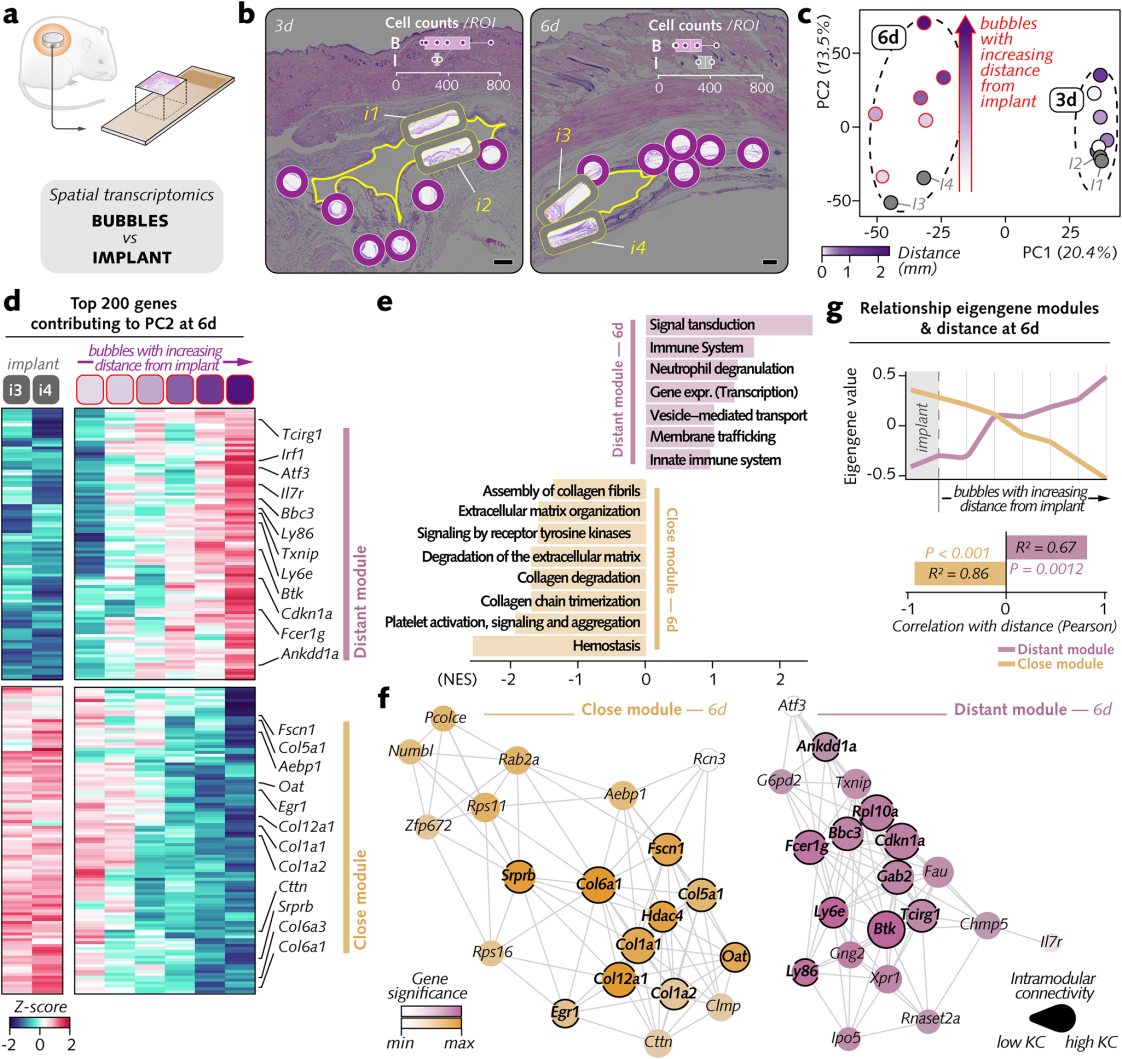
Spatial and temporal influence of implant proximity on transcriptomic regulation in peribubble regions. a) Schematic overview of spatial transcriptomics analysis comparing gene expression in peribubble regions with that at the implant interface at 3 and 6 d. b) Histological images illustrating the location of ROIs around bubbles and at the implant interface at 3 and 6 d. Cell counts per ROI and distance measurements from the implant interface are annotated. c) Principal component analysis (PCA) of gene expression data, showing separation along PC1 between 3 and 6 d, with PC2 capturing the influence of bubble distance from the implant at 6 d. Distance‐dependent clustering along PC2 indicates evolving transcriptomic patterns on the basis of spatial positioning relative to the implant (PC = principal component). d) Heatmap of the top 200 genes contributing to PC2 at 6 d, revealing two clusters with opposite expression patterns as the distance from the implant increased: “Distant module” and “Close module”. e) Pathway enrichment analysis of genes within the “Distant” and “Close” modules at 6 d was performed via *ReactomePA* and *clusterProfiler* and. f) Coexpression network analysis of the hub genes in the “Distant” (purple) and “Close” (orange) modules, highlighting genes with high gene significance (GS) and eigengene‐based connectivity (KC). g) Correlation (Pearson) of eigengene values with distance from the implant at 6 d, showing distinct trends for the “Distant” (immune‐related) and “Close” (matrix‐related) modules. Scale bar: b = 1 mm.

The influence of the bubble–implant distance on the transcriptomic regulation of cells near the bubbles suggested that this cellular response might be directly affected by the implant itself. To verify this assumption, we compared the gene expression profiles of cells in the peribubble region with those at the implant interface via spatial transcriptomics (**Figure** 5a). New ROIs were defined with cells ≈20 µm from the soft tissue‐implant interface and matched in terms of cell count to the peribubble ROIs (Figure **5**b). PCA (Figure 5c) revealed a clear separation between 3 and 6 d along PC1. Unlike at 3 d, the influence of distance from the implant became evident along PC2 at 6 d, with samples progressively shifting away from the implant region. This distance‐dependent clustering suggests that transcriptomic regulation evolves over time and becomes more spatially defined by 6 d.

The top 200 genes contributing the most to PC2 at 6 d (Figure 5d) illustrated the influence of the bubble‐to‐implant distance, with two distinct sets of genes showing opposite expression patterns as distance increased. Weighted gene coexpression network analysis (WGCNA) of these genes revealed two distinct modules on the basis of proximity to the implant: *i)* the *“Distant module”*, enriched with inflammation and immune response‐related markers such as *Btk, Cdkn1a, Tcirg1, Ly6e*, and *Ly86*; and *ii)* the *“Close module”*, dominated by matrix‐related genes, including *Col12a1, Col1a1, Col6a1, Col5a1*, and *Egr1* (Figure 5d). The distinct functional pathways corresponding to these modules were further confirmed by enrichment analysis at 6 d (Figure 5e). The *“Distant module”* was linked to immune and inflammatory responses, including pathways such as neutrophil degranulation and innate immune system activation. In contrast, the *“Close module”* was enriched for matrix‐related pathways, including collagen fibril assembly and extracellular matrix organization.

Further investigation into these modules identified key hub genes, which were highlighted by their central roles in the coexpression networks (Figure 5f). These genes exhibited both high gene significance (GS) and eigengene‐based connectivity (KC), reflecting their function as regulators of their respective gene programs. In the *“Distant module”*, the hub genes included *Btk*, a key regulator of B‐cell receptor and TLR signaling;^[^ ^34^ ^]^ *Cdkn1a* (p21), which is involved in the macrophage response;^[^ ^35^ ^]^ and *Ly6e*, which modulates both the innate and adaptive immune systems upon interferon stimulation.^[^ ^36^ ^]^ In the *“Close module”*, the hub genes were primarily involved in matrix assemblies, such as *Col1a1, Col6a1*, and *Col5a1*, as well as matrix regulation, such as *Hdac4* and *Egr1*.

The relationship between the eigengene values and distance from the implant clearly revealed a spatial influence on gene expression at 6 d (Figure 5g) but not at 3 d (Figure S12, Supporting Information). The *“Distant module”* showed a strong positive correlation between eigengene values and increasing distance from the implant (R^2^ = 0.67), indicating the upregulation of immune response genes as bubbles moved farther from the implant. Conversely, *“Close module”* displayed a strong negative correlation with distance (R^2^ = 0.86), indicating that matrix‐related gene expression was highest near the implant and decreased as the distance increased. This implant distance‐dependent behavior further reinforces the spatial segregation of immune activation and matrix organization in response to bubbles. Moreover, the spatial regulation also unfolds over time (Figure S13, Supporting Information), as evidenced by a more pronounced inflammatory (*Spp1, Ebi3, Stat5b, Apoe*, and *Stx11*) and mechanoregulatory (*F13a1*) signature at the bubble sites and by the marked extracellular matrix‐related upregulation (*Col1a1, Col4a1, Col5a1, Col6a1*, and *Acta2*) at the implant interface at 6 d compared to 3 d.

In conclusion, these results demonstrate that over time and with increasing distance from the implant, peribubble regions develop a proinflammatory transcriptomic signature markedly different from that at the implant interface. This spatial and temporal shift indicates that the direct influence of the implant on surrounding tissues diminishes as bubbles move farther away, leading to spatial segregation where localized immune activation occurs around the bubbles, whereas matrix organization activities are concentrated near the implant.

## 3 Discussion

The formation of gaseous cavities is a conspicuous feature of the healing process in tissues neighboring biodegradable magnesium implants in patients. These cavities arise when magnesium degrades in physiological fluids, releasing hydrogen gas. Although most of this gas diffuses into the bloodstream, an excess production that surpasses the tissue's clearance capacity leads to localized gas accumulation, a process similar to that observed in pulmonary emphysema or anesthesia bubbles.^[^ ^37^, ^38^ ^]^

**Figure 6:**
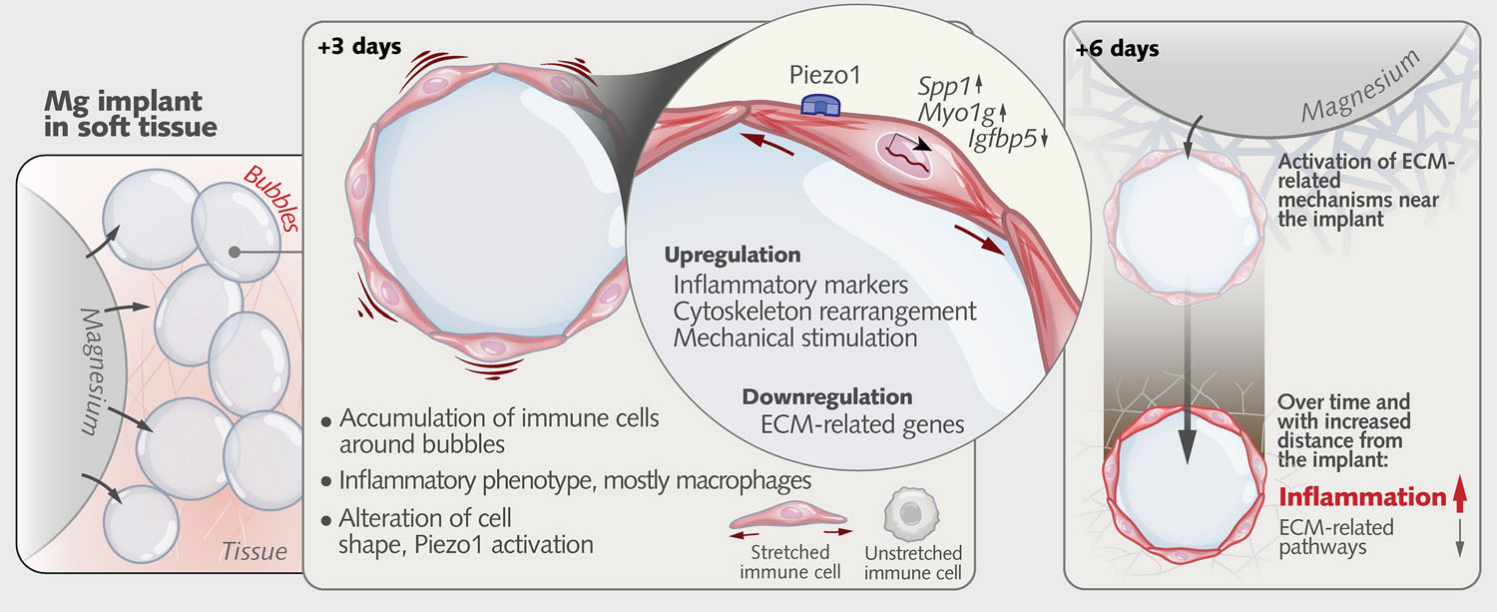
Schematic summary of cellular and molecular responses to bubbles from magnesium implants. Bubbles accumulate in soft tissue around magnesium implants, prompting robust immune cell infiltration–predominantly macrophages– and upregulation of inflammatory and mechanoregulatory markers (e.g., *Spp1*, *Myo1* *g*, and Piezo1) within the first few days. Meanwhile, extracellular matrix (ECM)‐related gene expression (e.g., *Igfbp5*) is suppressed at the bubble site but is enhanced near the implant interface, reflecting localized regenerative processes. Over time, particularly by 6 d, bubbles farther from the implant exhibit a stronger inflammatory signature, whereas regions closer to the implant show increased ECM remodeling. This highlights both the spatial and temporal segregation of immune activation and tissue organization in response to bubble formation.

However, the biological effects of these entities remain elusive. Whether these gas bubbles facilitate or hinder healing around magnesium implants has been a longstanding question among clinicians and materials scientists alike. To address this, we utilized spatially resolved transcriptomics to investigate the cellular response near bubbles and magnesium implants in vivo for the first time. Our data demonstrate that bubbles surrounding magnesium implants consistently induce an increased inflammatory response and trigger alterations in cell shape and cytoskeleton organization programs (**Figure** **6**). This reaction, however, appears to be spatially and temporally dependent; specifically, over time and with increasing distance from the implant, peribubble regions develop a proinflammatory transcriptomic signature markedly different from that at the implant interface, where extracellular matrix organization activities are concentrated. This increase in extracellular matrix‐related activities near the implant suggests a localized pro‐regenerative environment fostered by the implant itself. In contrast, the peribubble regions which are farther away lack this regenerative stimulus and instead exhibit heightened inflammation. Taken together, these spatial insights suggest that close monitoring of bubble formation in patients for potential complications until bubble‐free magnesium implants become a clinical reality is crucial.

Our data across several analytical modalities–including morphometry, immunohistochemistry, and detailed spatially resolved transcriptomic analysis—of the cells closest to the bubbles establish that these entities consistently induce, at both protein and gene levels, a robust proinflammatory response. This finding contradicts the notion that gas released from magnesium implants has an anti‐inflammatory effect^[^ ^39^ ^]^ and prompts questions into its role in the early inflammatory milieu around Mg implants.^[^ ^3^, ^5^ ^]^ The formation of gas bubbles may drive inflammatory cell recruitment through chemotactic gradients, as suggested by the upregulation of markers such as *Ccl5* and *Ccr5*.

Among the inflammatory transcripts upregulated by bubbles in their close vicinity, *Spp1* stood out as the gene with the most prominent expression. Secreted phosphoprotein 1 (SPP1) is a key regulator of hematopoietic stem cells and is closely associated with macrophages^[^ ^40^, ^41^ ^]^ in response to biomaterials implanted in soft tissues.^[^ ^28^, ^42^ ^]^ Single‐cell RNA sequencing and spatial transcriptomic studies have demonstrated an important role of SPP1 in wound healing upon biomaterial implantation via the activation of proliferative macrophages and foreign body response giant cells.^[^ ^28^, ^42^ ^]^ In addition, in pathological contexts such as obesity‐related inflammation, SPP1 serves as a pivotal mediator by acting in a self‐reinforcing manner to promote further macrophage recruitment and amplification of the inflammatory response.^[^ ^43^ ^]^ Therefore, the strong enrichment of macrophages in the bubble microenvironment, as evidenced by cell type deconvolution via spatial transcriptomics and immunohistochemistry, confirms the key role of these inflammatory cells in integrating the signals from the bubbles.

While SPP1 is also often suggested to promote the polarization of macrophages toward the proliferative M2 subtype, emerging evidence indicates that proinflammatory M1 macrophage subtypes might also be linked to elevated levels of SPP1.^[^ ^44^ ^]^ Our results support the latter notion by revealing strong immunoreactivity of the proinflammatory macrophage markers iNos and Cd68 together with a pronounced proinflammatory gene signature and activation of related pathways around the bubbles. For example, proinflammatory genes such as Interferon regulatory factor 7 (*Irf7*) or Cytochrome b‐245 beta chain (*Cybb*) are strongly expressed near bubbles. CYBB encodes the key superoxide‐generating enzyme NADPH in macrophages and is associated with increased oxidative stress in these cells^[^ ^45^ ^]^ in response to implants.^[^ ^46^ ^]^ IRF7 is the “master regulator” for the production of type I interferon and, together with other interferon‐regulated genes, plays an important role in the proinflammatory response of M1 macrophages.^[^ ^47^ ^]^ IRF7 appears to expand to control the release of extracellular vesicles to influence the behavior of cells in the tumor microenvironment.^[^ ^48^ ^]^ Therefore, whether cell‒cell communication mediating extracellular vesicles, eventually via IRF7, might be involved in the response to bubbles remains to be elucidated.

Cells and tissues are biological materials that sense and respond to their environment.^[^ ^49^ ^]^ The accumulation of gas released from the implant to form bubbles implies a change in the mechanical state of the surrounding tissues. In this study, we demonstrate for the first time that, in response to bubbles, tissue deformation, changes in cellular shape, and pronounced alterations in transcriptomic programs related to cytoskeletal rearrangement occur in the bubble microenvironment. Taken together, these observations suggest that tissues near bubbles undergo a process of tissue fluidization^[^ ^50^, ^51^ ^]^ where mechanical forces induce cells to reorganize their cytoskeleton and adapt their behavior to the changing environment, such as in the context of wound healing^[^ ^52^ ^]^ or cancer progression.^[^ ^53^, ^54^ ^]^ This cellular rearrangement is reflected in our study by the pronounced upregulation of the *Myo1* *g, Dock8*, and *Vav3 genes*, which are required for immune cell elasticity via actin network deformation.^[^ ^29^, ^32^, ^55^, ^56^, ^57^ ^]^ Similarly, the elevated immunodetection of the mechanosensitive ion‐channel Piezo1 further supports the notion that mechanical forces exerted by bubbles influence cellular behavior. Piezo1, which is activated by mechanical stimuli, facilitates cation influx and triggers downstream signaling pathways.^[^ ^27^ ^]^ This mechanosensation is linked to inflammation, as the suppression of Piezo1 has been shown to reduce leukocyte recruitment.^[^ ^58^ ^]^ Therefore, the finding that cell densities around the bubbles were even greater than those at the tissue–implant interface raises the question of whether the mechanical cues from the bubbles and the resulting alteration in the mechanical state of the surrounding tissue might create a mechanical gradient that acts as a guidance cue to the cells.^[^ ^59^ ^]^

A crucial factor influencing the cellular response around the bubbles was the distance between the bubbles and the implant. We observed that as the bubble distance from the implant increased, the regenerative processes promoted by the implant diminished, allowing inflammatory responses to predominate around bubbles farther from the implant. This dichotomy is exemplified by the robust repression of the profibrotic gene *Igfbp5* near the bubbles, in contrast with the central regulatory role of several collagen‐related genes revealed by network analysis closer to the implant. Such contrasting effects of the implant and the bubbles could be mapped only through spatially resolved techniques such as spatial transcriptomics^[^ ^60^ ^]^ used in our study.

Importantly, this spatial discrepancy became particularly evident at 6 d, unlike at 3 d. The spatial variation increased over time as the healing process transitioned from the initial inflammatory stage to the subsequent regenerative phase following magnesium implantation. Therefore, the information transmitted from bubbles to surrounding cells varies across both biological and time scales.

These novel insights raise important questions about the spatial and temporal fate of bubbles generated by magnesium implants in adjacent tissues. Does the gas composition inside the bubbles influence at all the behavior of the surrounding cells? While our study focuses on the cellular and molecular responses to bubbles, the exact gas composition within these cavities remains to be fully elucidated. Although historical and theoretical evidence suggests a mixed gas environment rather than pure hydrogen,^[^ ^23^ ^]^ advancements in non‐invasive, in situ sensor technologies are needed for comprehensive gas profiling inside the bubbles.

Moreover, how do the bubbles evolve within their tissue microenvironment? Do small bubbles coalesce to form larger bubbles, or do they dissipate individually? Are bubbles predisposed to migrate deeper into the tissues over time? What mechanisms enable the tissues to resorb within a few weeks the large number of bubbles formed just days after magnesium implantation? Do smaller bubbles move more easily through tissue structures? What are the local versus systemic implications of this bubble formation—do bubbles primarily impact the immediate environment, or could they initiate broader responses?

Given that the evolution of bubbles was not tracked in real time in tissues, our study does not address these questions. This limitation highlights the need for further studies employing advanced imaging methods and innovative approaches to monitor bubbles in vivo.^[^ ^61^, ^62^ ^]^ Nevertheless, from a translational perspective, our findings emphasize the importance of closely monitoring bubble formation in patients, especially those immunocompromised or stented with biodegradable magnesium implants. For these patients, developing magnesium implants with limited gas release for a controlled inflammatory response becomes essential to secure implant integration and minimize potential complications. To achieve this, optimizing implant formulation through alloying, together with the implementation of effective non‐invasive, real‐time in situ monitoring of gas release from magnesium implants will be crucial.

## 4 Conclusion

This study demonstrated that bubbles generated by magnesium implant degradation, in addition to mere reservoirs of gas, play a more intricate role than previously believed. Tissue and cells respond to nearby bubbles through pronounced inflammation and morphological alterations via cytoskeletal rearrangement, with a severity varying depending on time and distance from the implant. These findings suggest that when bubbles accumulate and persist in the soft tissue adjacent to magnesium implants, they might sustain inflammation and tissue deformation, thereby impeding regenerative processes and encouraging complications. This emphasizes the need for the design of magnesium implants with controlled gas release and for meticulous clinical monitoring of bubble evolution in patients, particularly those with compromised immune responses.

## 5 Experimental Section

### Implants

High‐purity magnesium (99.998%) implants were fabricated from cylindrical ingots cast via a modified permanent direct chill casting technique under an Ar atmosphere containing 3 vol.% SF₆.^[^ ^63^, ^64^ ^]^ Briefly, magnesium (Magontec) was melted at 720 °C and then poured into steel molds preheated to 700 °C. After holding for 2 min, the melt was directionally water‐quenched by lowering the molds into a water bath. Composition analysis by atomic absorption spectroscopy and spark emission spectroscopy revealed the final elemental contents to be Mg (balance, 99.995 wt.%), Fe (0.0048 ± 0.0001 wt.%), Cu (0.0003 ± 0.0001 wt.%), and Ni (< 0.0002 wt.%). Each cast ingot (≈ 49 mm in diameter, 150 mm long) was prepared for indirect extrusion by preheating to 350 °C for 1 h. The extrusion process was performed using a horizontal press (maximum force 2.5 MN, Müller Engineering GmbH) at a ram speed of 2.4 mm s^−1^, producing a round bar with a 10 mm diameter (extrusion ratio 1:25). The extruded bar was then machined down to 9 mm in diameter and sectioned into discs ≈1.5 mm in thickness using a side milling cutter. To achieve a uniform surface finish, discs were polished with 2500‐grit SiC paper and cleaned ultrasonically in n‐hexane, acetone, and 100% ethanol sequentially. Finally, the discs were air‐dried under vacuum, packaged individually, and sterilized by gamma irradiation (30.2 kGy, BBF GmbH).

The surface of cleaned and sterilized magnesium implants (n = 4) was examined by scanning electron microscopy (SEM; SU‐8000, Hitachi) in backscattered electron (BS‐SEM) and secondary electron (SE‐SEM) modes at an accelerating voltage of 15 kV. Elemental composition was analyzed using energy‐dispersive X‐ray spectroscopy (EDX; UltraDry EDS Detector, NSS v.3.2, Thermo Fisher Scientific). Cross‐sections for microstructure analysis were prepared with metallographic cutting equipment (MICRACUT 151, Metkon), using a glycerol‐in‐ethanol coolant (1:3 v/v). Polished sections of magnesium discs were subsequently ion milled (IM 4000, Hitachi) before BS‐SEM imaging at 15 kV.

### Animal Surgery

Animal experiments were conducted as previously described,^[^ ^5^ ^]^ following the ARRIVE guidelines and approved by the Local Ethical Committee for Laboratory Animals at the University of Gothenburg, Sweden (Dnr‐02437/2018). Briefly, male Sprague–Dawley rats (300–380 g; Taconic Biosciences) were anesthetized by inhalation of 4% isoflurane, and subcutaneous pockets were formed on the dorsum into which the magnesium discs were inserted following a randomization scheme. Sham‐operated sites were prepared similarly without implant placement. All wounds were closed with sutures, and animals received analgesia (Temgesic, 0.03 mg kg^−1^). At 1 , 3 , 6 , and 28 d post‐surgery, the rats were euthanized by an overdose of pentobarbital. Subcutaneous discs, with or without surrounding soft tissues, were harvested and fixed in formalin. In addition, 6 mm punch biopsies of tissues adjoining the implants were collected and placed into a preservation medium (RNA Shield, Zymo Research) for subsequent molecular analyses.

### Histology

Samples of peri‐implant soft tissue (n = 8 per group per time point) were fixed in formalin, dehydrated, and embedded in paraffin. Serial sections were then cut to a thickness of 5 µm (Leica RM 2255, Leica Biosystems Nussloch GmbH) and deparaffinized in xylene prior to staining with hematoxylin and eosin. Additional 15–20 µm thick sections obtained from LR White‐embedded samples (implant and per‐implant tissues) were stained with toluidine blue (1%). Histological examination was conducted with an optical microscope (Nikon Eclipse E600, Nikon).

### Immunohistochemistry

Sections of peri‐implant soft tissue were deparaffinized, rehydrated, and washed in PBS. The sections were then incubated at 90 °C for 20 min for antigen retrieval, followed by 30 min of blocking with 5% goat serum in 4% bovine serum. Primary rabbit polyclonal antibodies against iNos (PA1036, Thermo Fisher), Cd68 (PA581594, Thermo Fisher), Mrc1 (PA5101657, Thermo Fisher), and Piezo1 (PA5‐106296, Thermo Fisher) were diluted to appropriate concentrations (Table S1, Supporting Information), and the sections were incubated in the antibody solutions for 2 h at room temperature. For immunoreactivity detection, a horseradish peroxidase detection system (Pierce Horseradish Peroxidase, Thermo Fisher) was used with DAB (Metal Enhanced DAB Substrate Kit, Thermo Fisher) as the substrate following the manufacturer's instructions. Negative control sections were prepared according to the same protocol but without primary antibodies.

### Image Analysis

Full‐slide scans of all immunostained sections were acquired with a Plan Apo 20×/0.75 objective using imaging software (NIS‐Elements, Nikon) and then imported into QuPath software (v.4.3^[^ ^65^ ^]^) for image analysis. Identical settings and thresholds were applied to all the acquired scans. The peri‐implant bubbles were manually outlined (wand tool) on the scanned sections, and their morphometry was analyzed according to the following parameters: area, perimeter, distance (from the bubble centroid) to the interface with the implant, aspect ratio, and circularity. The peribubble cellularity was subsequently analyzed in regions of interest (ROIs) encompassing tissue within 20 µm of the bubble outline to calculate the density of total cells and the proportion of protein‐expressing cells via the automated “positive cell detection” plugin. The cell and nucleus circularity, total cell density, and positive cell proportion in the peri‐bubble ROIs (“*Bubble*”) were compared to those at the interface of the implant–soft tissue (“*Implant*”) within 20 µm from the interface with the implant in the same section (Figure S2, Supporting Information).

Additional circular control ROIs (“*Control*”) were delimited in the subcutaneous loose connective tissue of soft tissue sections ≈1 mm from the overlaying panniculus carnosus muscle and >1 mm lateral to the implant interface. The areas of these control ROIs were defined as the average bubble area calculated in soft tissue (45 000 µm^2^).

### Quantitative Polymerase Chain Reaction (qPCR)

qPCR was performed on punches sampled from soft tissues that interfaced with the implants and from sham sites. The samples were homogenized in RNA Shield to an aqueous phase via a TissueLyser instrument (Qiagen GmbH) and frozen (−80 °C). Then, RNA was extracted from the cells in all the samples via RNeasy Micro Kit (Qiagen GmbH) following the manufacturer's instructions. A pilot study representative of the present experiments allowed the verification of RNA quality (Pico 6000 RNA Kit in Bioanalyzer 2100 electrophoresis system, Agilent Technologies) and RNA concentration (Nanophotometer P‐36, Implen GmbH) in the tissue samples. Reverse transcription into cDNA was carried out via the use of the TATAA GrandMaster cDNA Synthesis Kit (TATAA Biocenter AB) following the manufacturer's instructions. Predesigned and validated primers were purchased from Bio‐Rad (Table S2, Supporting Information). The genes of interest were *Spp1, Myo1* *g, Irf7*, and *Cybb*. The reference gene panel included the following rat genes: *Actb*, *Gapdh*, and *Hprt1* (Bio‐Rad), and gene stability was determined via GeNorm and NormFinder (GenEx software, Multid). The most stable reference gene expression was achieved for the combination of *Hprt1* and *Gapdh*, which was used for normalization. Normalized relative quantities were calculated via the delta–delta–Cq method.

### NanoString GeoMx Digital Spatial Profiling (DSP)

For spatial transcriptomics analysis, the NanoString GeoMx DSP platform was used in accordance with protocols provided by NanoString Technologies:

### Slide Preparation and Sample Collection

Sections of 5 µm thickness were prepared from the FFPE samples at 3 and 6 d, mounted onto SuperFrost Plus slides. The sections were then deparaffinized and hydrated, followed by antigen retrieval using Tris‐EDTA pH 9.0 for 25 min at ≈99 °C in a steamer. Subsequently, RNA targets were exposed using proteinase K followed by a post‐fixation step using 10% neutral buffered formalin for 5 min to preserve morphology. Probes targeted to the whole mouse transcriptome (corresponding to ≈21 000 transcripts; GeoMx Mouse Whole Transcriptome Atlas) were hybridized to the slides overnight, followed by multiple stringency washes to minimize background signal. To aid in the selection of ROIs, slides were stained with antibody anti‐Collagen VI clone EPR17072 fluorescently conjugated to Alexa Fluor‐ 488 (ab200429; 1:100 dilution; Table S1, Supporting Information) for extracellular matrix visualization, and Syto83 dye to stain nuclei. Following staining, slides were scanned on a GeoMx Digital Spatial Imager (NanoString) and ROIs were selected. A total of 24 ROIs were selected similar to the ROIs assigned to image analysis from full‐slide scans of the immunostained sections: *i)* 12 “*Bubbles*” ROIs outlining 12 selected bubbles, with a thickness of 20 µm, located at different distances from the implant–soft tissue interface; *ii)* 8 circular “*Controls*” ROIs in the subcutaneous loose connective tissue ≈1 mm from the overlying panniculus carnosus muscle and >1 mm lateral to the implant interface; and *iii)* 4 “*Implant*” ROIs within 20 µm from the interface implant–soft tissue. All the ROIs were selected via geometric tools. Each ROI was collected into a single well in a 96‐well plate.

### GeoMx Sequencing

Next‐generation sequencing libraries were generated for each ROI. The resulting libraries were subjected to quality control by DNA Qubit and TapeStation to determine library yield and size, respectively. Finally, libraries were diluted and loaded on a 25 B flow cell of an Illumina NovaSeq X Plus sequencer in a 2 × 150 bp configuration. A total of ≈429 million raw reads with UMIQ30 scores >0.997, sequencing saturation >95%, and an alignment rate of 70–95%. The raw sequencing data (.fastq files) was bioinformatically trimmed prior to data processing into digital count conversion (DCC) files via the NanoString GeoMx NGS Pipeline 2.3.3.10, with effective UMI deduplication and read pair merging.

### Normalization and Scaling of GeoMx Count Data

Quality control (QC) was implemented via the following thresholds: minimum raw reads = 1000; aligned read threshold = 80%; stitched read threshold = 80%; trimmed read threshold = 80%; sequencing saturation threshold = 50%; geometric mean of negative probe counts ≥10; and nuclei count threshold ≥200. Further filtering was applied to exclude segments with low probe performance, using a probe geomean ratio threshold of 0.1 and a Grubbs test threshold for 20% of the ROIs. Expression filtering was conducted using the higher value between the limit of quantification (LOQ) and a user‐defined threshold of 2. Quality control was performed according to the default parameters recommended by NanoString. For normalization, the third quartile (Q3) method was employed, whereby each gene's expression was normalized by dividing by the 75th percentile of expression in each sample, followed by scaling across all samples. This approach eliminates differences in counts between samples owing to ROI‐specific properties such as size and RNA‐binding efficiency. Moreover, the background signal was estimated using the geometric mean of the negative control probes. A defined set of reference genes was used for consistent target filtering across samples.

### Data Analysis for Spatial Transcriptomics

The downstream analysis of spatial transcriptomics was performed with R v4.4.1. Differential expression analysis (DEA) was conducted using a linear mixed model (LMM) to account for repeated measurements within the same slide. For comparisons between *“Bubbles”* and “*Controls*” ROIs, both a random intercept and a random slope were included, while for comparing *“Bubbles”* versus “*Implant*”, only a random intercept was used. The Bonferroni‐Hochberg method was applied to adjust *p*‐values for multiple testing, and genes with fold changes corresponding to *p* < 0.05 were considered significant. The following R packages were employed: lme4 (v1.1‐30), lmerTest (v3.1‐3), emmeans (v1.8.1), and parallel (v4.2.3).

Pathway analysis was performed using the ReactomePA (v1.42.0) and clusterProfiler (v4.6.2) packages, incorporating Reactome Database Build 78 + NCBI_0 812 2021. Enrichment analysis was conducted for the significant (*p* < 0.05) differentially expressed genes, focusing on pathway‐level and functional interpretations within the Reactome database. The analysis utilized gene set enrichment analysis (GSEA) implemented with the fGSEA package.^[^ ^66^ ^]^

Cell type deconvolution was conducted to estimate cell type abundance within ROIs with the SpatialDecon (v1.0) algorithm,^[^ ^67^ ^]^ using the Mus Immune Family profile matrix (https://github.com/NanoString‐Technologies/GeoScriptHub).

Weighted gene co‐expression network analysis (WGCNA) was performed using the WGCNA (v1.71) package^[^ ^68^ ^]^ to identify co‐expression modules and eigengene associations. Correlations between module eigengenes and biological distances were analyzed using Pearson's correlation, with statistical significance assessed using *p*‐values derived from Student's t‐distribution.

Data visualization was conducted using ggplot2 (v3.4.2), ggrepel (v0.9.3), pheatmap (v1.0.12), viridis (v0.6.2), gridExtra (v2.3), and igraph (v1.2.9) for creating volcano plots, heatmaps, and network graphs.

### Electron Microscopy

Secondary electron scanning electron microscopy (SE‐SEM) was used to examine the surface of the implants. Implants were retrieved from subcutaneous pockets without the associated tissues, fixed in 70% methanol (6 min at −20 °C), dehydrated in a graded ethanol series, and allowed to air‐dry overnight. Au sputter‐coated samples were then observed under an Ultra 55 FEG SEM (Leo Electron Microscopy Ltd.) operated in secondary electron mode at an accelerating voltage of 5 kV at a working distance of 5 mm.

### Statistical Analysis

Statistical analysis of all non‐transcriptomic data (bubble morphometry, immunohistochemistry quantifications, qPCR data) was performed in SPSS (v.27, IBM Corporation). The nonparametric tests Kruskal‒Wallis and Mann‒Whitney *U* tests were used for unpaired comparisons. In addition, the nonparametric tests Friedman's two‐way ANOVA by rank and Wilcoxon signed‐rank test were used for paired comparisons. Spearman's rank correlation was used to assess associations between variables. Differences with *p* < 0.05 were considered statistically significant.

## Conflict of Interest

The authors declare no conflict of interest.

## Author Contributions

H.B.A. performed conceptualization, methodology, investigation, visualization, and wrote the original draft, also wrote the manuscript reviewed and edited the file, and acquired funding; J.P. performed investigation, wrote the original manuscript, and reviewed and also edited the file; O.O. performed conceptualization, methodology, investigation, supervision, and wrote the original manuscript and reviewed and also edited the file; P.T. performed conceptualization, methodology, investigation, supervision, wrote the original draft, wrote, reviewed, and editing the manuscript and also acquired funding.

## Supporting information



Supporting Information

## Data Availability

The data that support the findings of this study are available from the corresponding author upon reasonable request.
