# Supplementary material for: Gas Bubbles from Biodegradable Magnesium Implants Convey Mechanical Cues and Promote Immune Cell Stimulation
Source: Adv Sci (Weinh). 2025 May 11;12(28):2503123. doi: 10.1002/advs.202503123 (PMC12302579; doi:10.1002/advs.202503123)
Supplement: Supplementary file 1 — Supporting Information [file ADVS-12-2503123-s001.docx]

Supplementary material

**Gas bubbles from biodegradable magnesium implants convey mechanical cues and promote immune cell stimulation**

*Heithem Ben Amara^1*^, Jincy Philip^1^, Omar Omar^2^, Peter Thomsen^1,2*^*

**Affiliations**

^1^ Department of Biomaterials, Institute of Clinical Sciences, Sahlgrenska Academy,

University of Gothenburg, Sweden.

^2^ Department of Biomedical Dental Sciences, College of Dentistry, Imam Abdulrahman bin Faisal University, Dammam, Saudi Arabia.

***Corresponding authors:**

Heithem Ben Amara (*ORCID:* ***0000-0002-7927-9838*)** & Peter Thomsen (*ORCID:*

***0000-0003-3910-6665*)**

Department of Biomaterials, Institute of Clinical Sciences

Sahlgrenska Academy, University of Gothenburg

E-mail: heithem.ben.amara@biomaterials.gu.se; peter.thomsen@biomaterials.gu.se

Box 412; SE-405 30

Gothenburg, Sweden


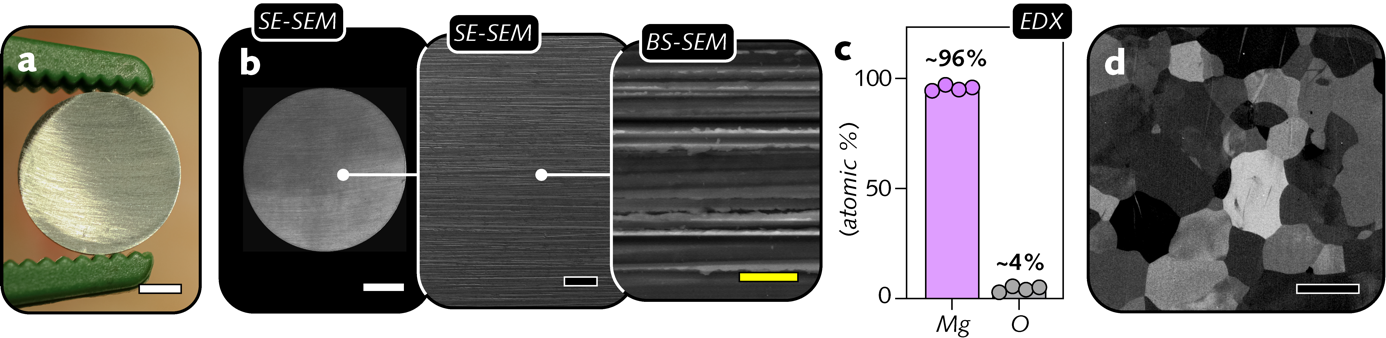


***Figure S1:* Characterization of the implants**

**a,** Disc-shaped magnesium implant (9 mm diameter, 1.4 mm thick) prior to insertion in the surgical sites in the animals. **b,** Scanning electron microscopy (SEM) in secondary (SE) and backscattered (BS) electron modes of the implant surface. **c,** Energy-dispersive X-ray spectroscopy (EDX) of the implant surface (n = 4). **d,** Cross-sectional BS-SEM image depicting the fine grain-sized microstructure of the implant.

Scale bar: **a** = 2 mm; **b:** *white =* 2 mm, *black =* 50 µm, *yellow =* 20 µm; **d** = 50 µm.


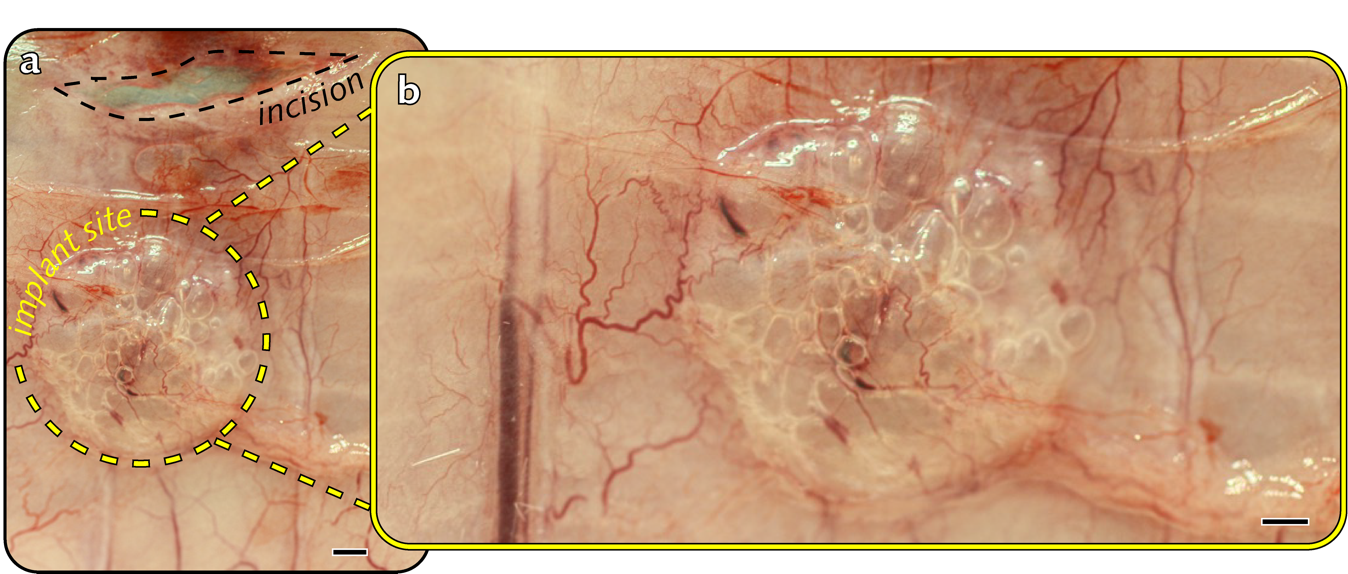


***Figure S2:* Bubbles in the soft tissue upon implantation with magnesium**

**a,** Photograph of soft tissue dissected from the back of a rat 3 d post-implantation of a magnesium disc (highlighted with a yellow broken line). The implantation site was accessed through the incision site (indicated by a black broken line). **b,** Magnified view of the implantation site showing multiple gas bubbles in the peri-implant tissue. Scale = 1 mm.


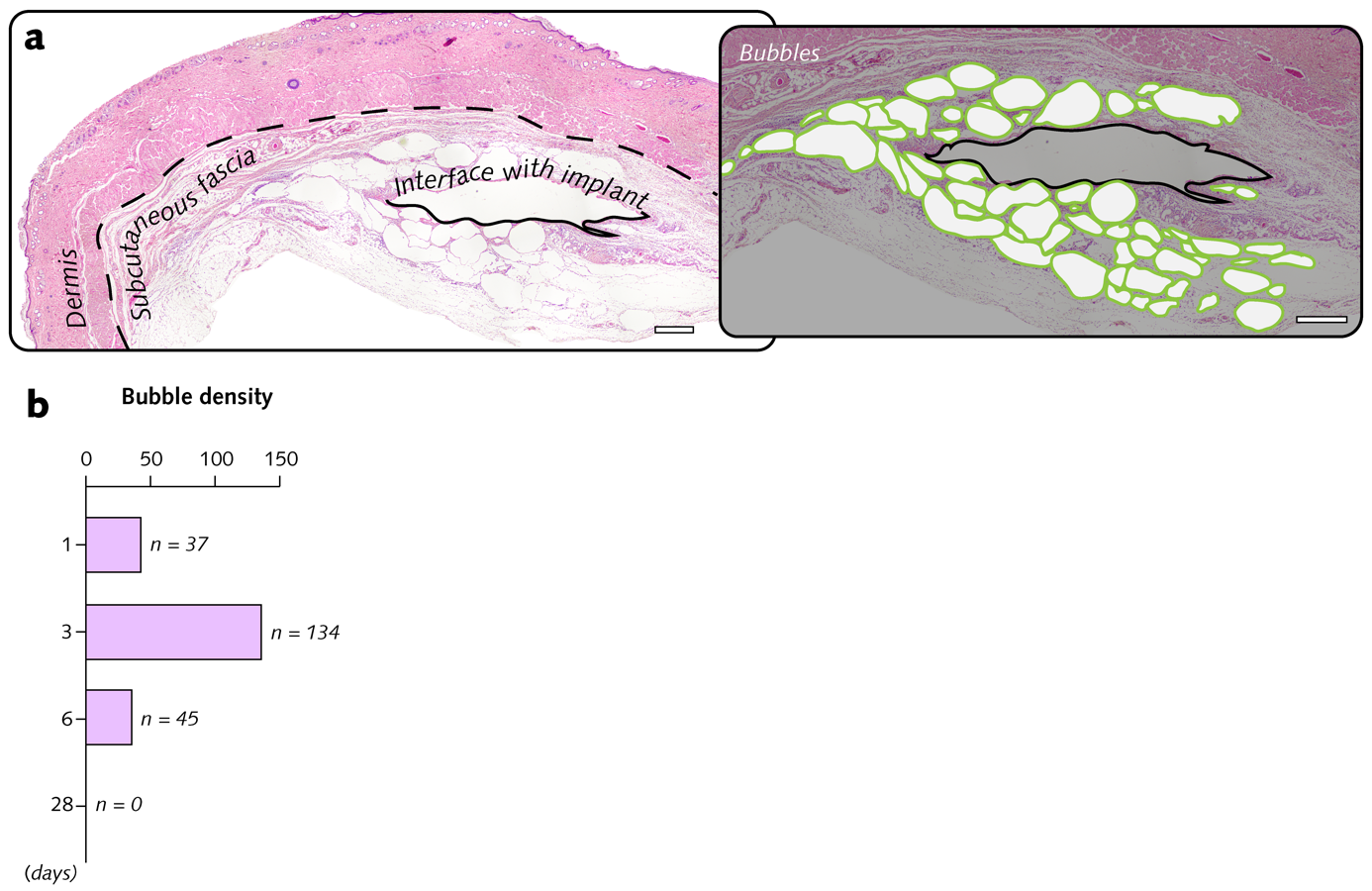
***Figure S3:* Density of bubbles in the peri-implant tissues**

**a,** Histological section of soft tissue 3 d after implantation of a magnesium disc in the subcutaneous fascia. The magnified micrograph (right) highlights gas bubbles (outlined in green) and their spatial relationship with the tissue–implant interface. The implant was collected from the tissue prior to tissue retrieval. Hematoxylin and eosin staining. **b,** Quantification of bubble density across all animals at each time point (n = 6 rats/time point). Scale = 500 µm.***
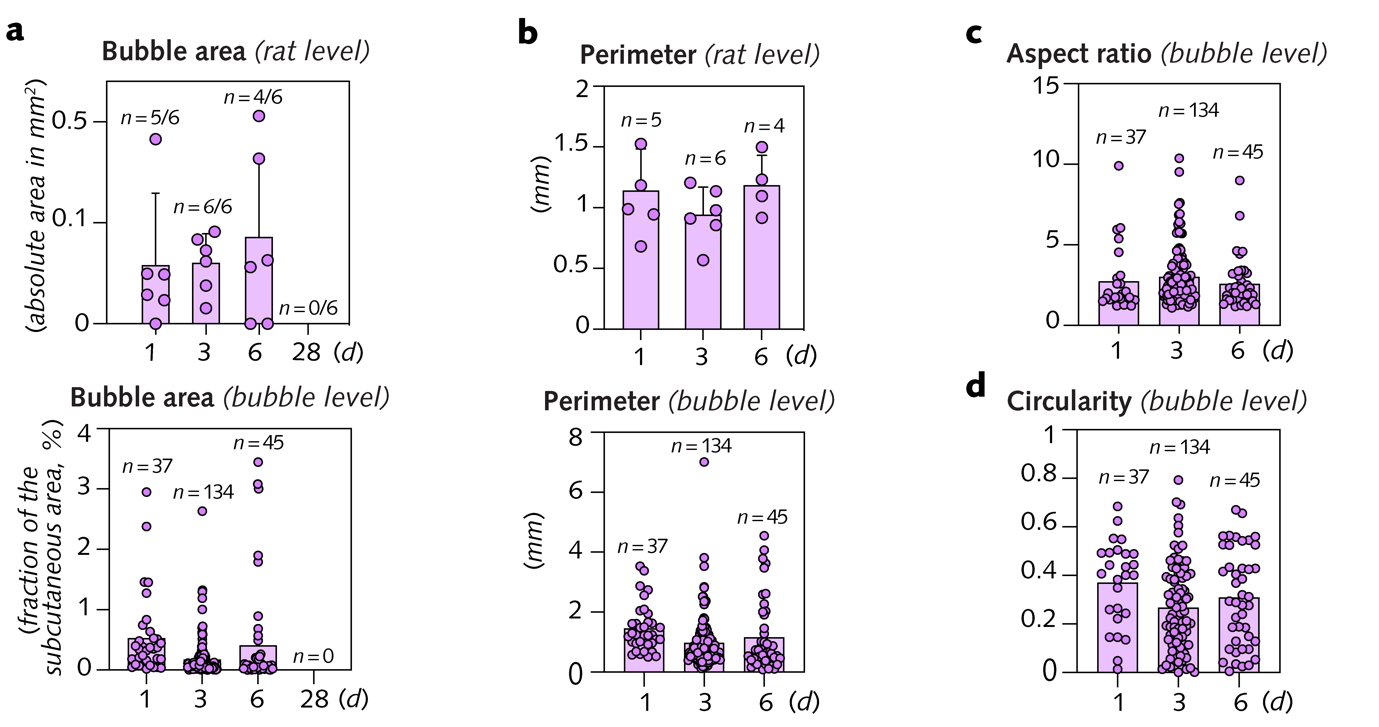
***

***Figure S4:* Area, perimeter, aspect ratio, and circularity of bubbles**

**a,** Bubble area shown as the absolute summed-area relative to the total subcutaneous fascia area in each rat (top; n = 6/group/time point) and as the fractional area of individual bubbles relative to the subcutaneous fascia (bottom; individual bubbles pooled from all analyzed tissue sections per time point). **b,** Bubble perimeter shown as the average perimeter per rat (top; n = 5–6/group/time point) and the individual bubble perimeter pooled across tissue sections (bottom). **c,** Aspect ratio of bubbles pooled across all tissue sections at each time point. **d,** Circularity of bubbles pooled across all tissue sections at each time point.

The data are shown as the means with error bars indicating the s.d.


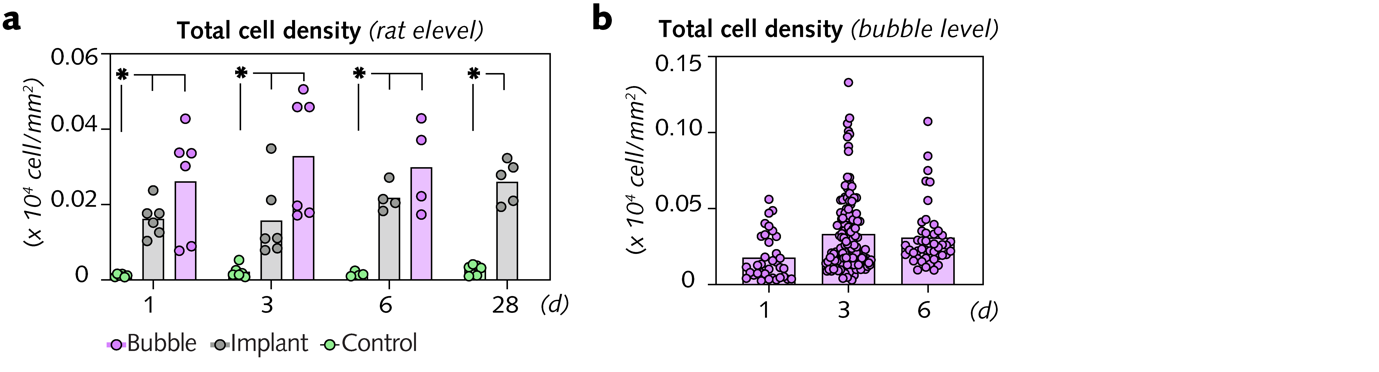


***Figure S5:* Density of cells around the bubbles**

**a,** Total cell density around shown as the average cellular density per rat in three regions: around bubbles (*Bubble,* purple), at the implant–tissue interface (*Implant,* gray), and in the control region (*Control,* green) (n = 6/group/time point). **b,** Total cell density around all bubbles pooled across tissue sections (n = 37 at 1d, n = 134 at 3 d, and n = 45 at 6 d).

The data are shown as the means. Friedman’s two-way ANOVA by rank test for paired comparisons of Bubble, Implant, and Control groups. Kruskal‒Wallis test for comparisons between time points.


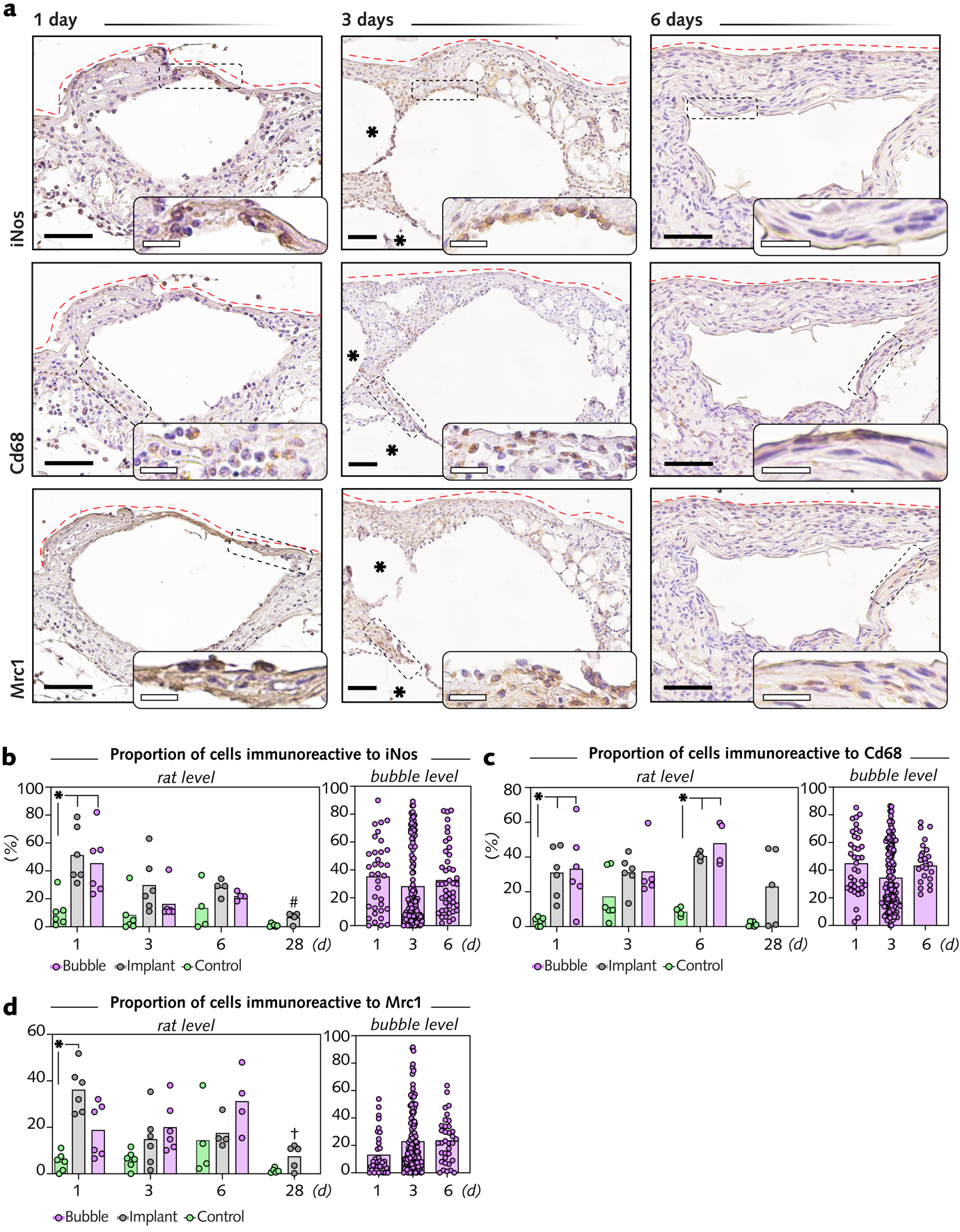


***Figure S6:* Immunoreactivity of inflammatory markers in cells around bubbles**

**a,** Immunostaining of iNos (top row), Cd68 (middle row), and Mrc1 (bottom row) in soft tissue sections at 1, 3, and 6 d after implantation with magnesium. Regions near the bubbles (asterisks) and the implant interface (red broken lines) are shown, with magnified insets highlighting immunopositive cells. **b-d,** Quantification of the proportion of cells immunoreactive to iNos (**b**), Cd68 (**c**), and Mrc1 (**d**) with average fractions per rat (left, n = 6/group/time point) and fractions in individual bubbles pooled across tissue sections level (right, n = 37 at 1d, n = 134 at 3 d, and n = 45 at 6 d) (Bubble region: purple, Implant region: gray, and Control region: green).

The data are shown as the means. Friedman’s two-way ANOVA by rank test for paired comparisons of Bubble, Implant, and Control groups. Kruskal‒Wallis test for comparisons between time points. * *P<*0.05, Bubble *versus* Control or Bubble *versus* Control. **#** *P<*0.05, 28 d *versus* 1 d and 3 d. **** *P<*0.05, 28 d *versus* 1 d.

Scale bars: **a**: *black*= 200 µm, *white=* 50 µm.

***
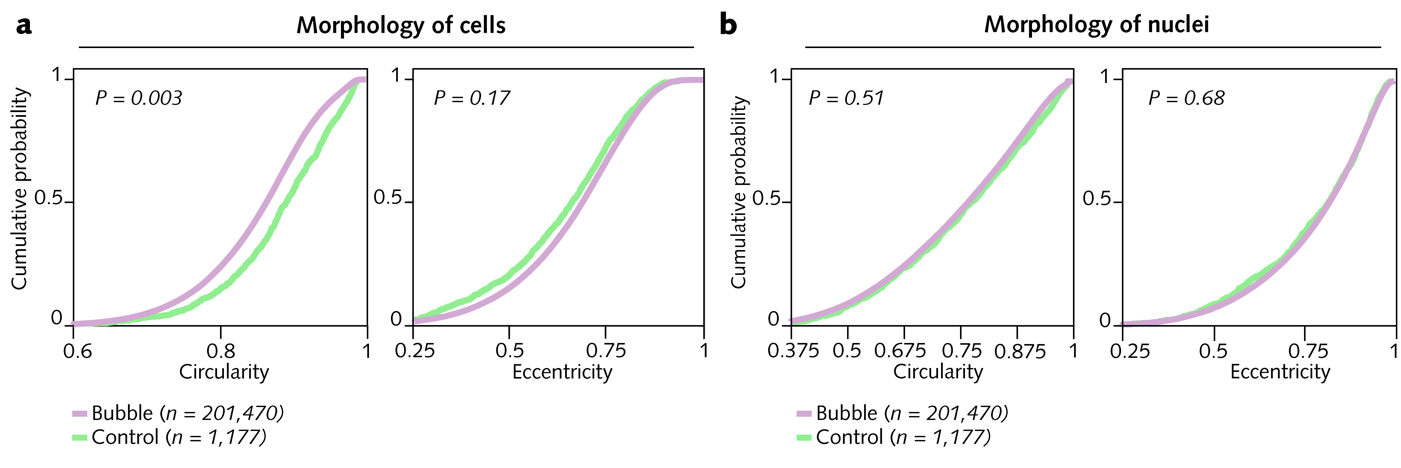
***

***Figure S7:* Morphology of the cells and their nuclei around the bubbles**

**a,** Cumulative probability distributions for cell circularity and eccentricity in bubble regions *versus* control regions. **b,** Cumulative probability distributions for nuclear circularity and eccentricity in Bubble regions *versus* Control regions. Wilcoxon rank-sum test.

***
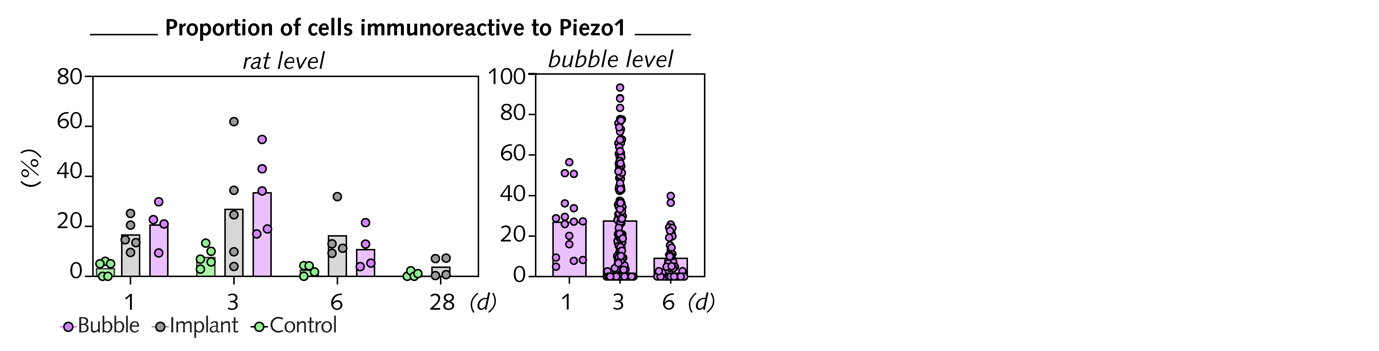
***

***Figure S8:* Immunoreactivity to the mechanosensitive ion channel Piezo1**

**a,** Quantification of the proportion of cells immunoreactive to Piezo1 with average fractions per rat (left, n = 6/group/time point) and fractions in individual bubbles pooled across tissue sections level (right, n = 37 at 1d, n = 134 at 3 d, and n = 45 at 6 d) (Bubble region: purple, Implant region: gray, and Control region: green).

The data are shown as the means.


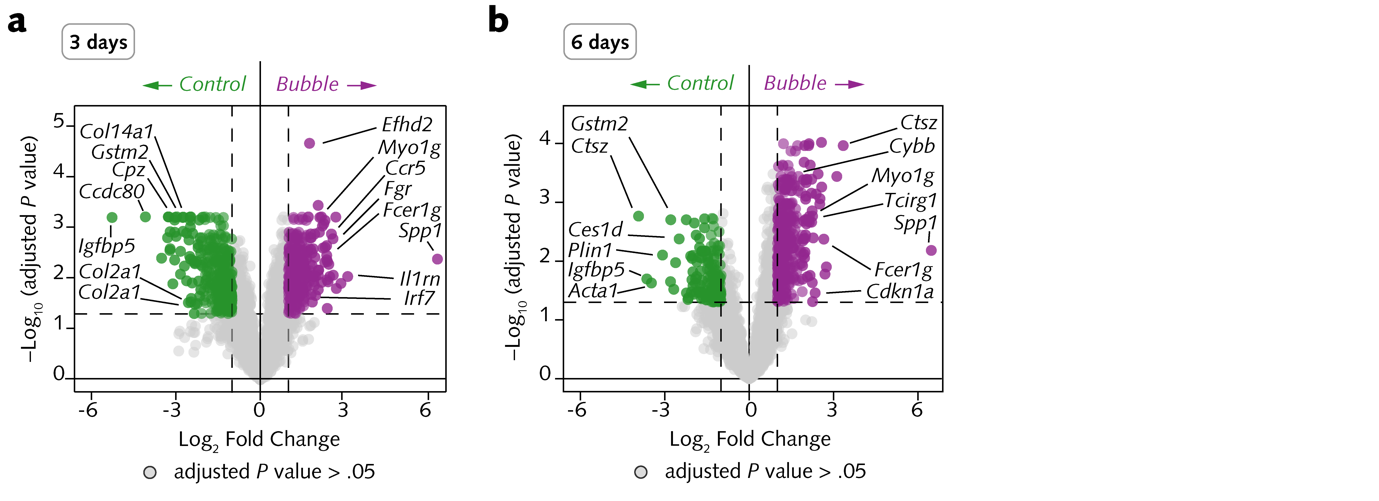
***Figure S9:* Differential gene expression between the Bubble and Control groups at 3 days and 6 days**

**a,** Volcano plots depicting the fold changes and significance of differentially expressed genes (DEG) in the Control (left) and Bubble (right) groups at 3 d. **b,** Volcano plots depicting the fold changes and significance of DEGs in the Control (left) and Bubble (right) groups at 6 d. Differential expression analysis (DEA) was conducted via linear models. Statistical significance: *P* < 0.05 was adjusted via the Benjamini‒Hochberg procedure.


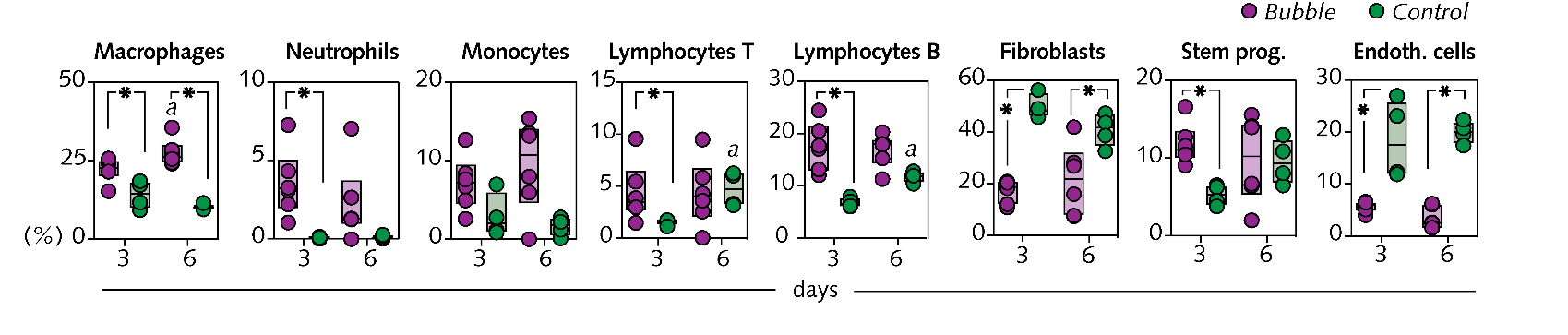
***Figure S10:* Deconvoluted cell types in the bubble and control groups**

Fraction of some cell types deconvoluted upon analysis via the *SpatialDecon* package. (Endoth = Endothelial cells; Stem prog = Stem and progenitor cells). * *P <* 0.05, Bubble *versus* Control. **a,** *P <* 0.05, 3 d *versus* 6 d in the Bubble or Control groups.

The data are shown as the means. Unpaired Mann‒Whitney *U* test.

***
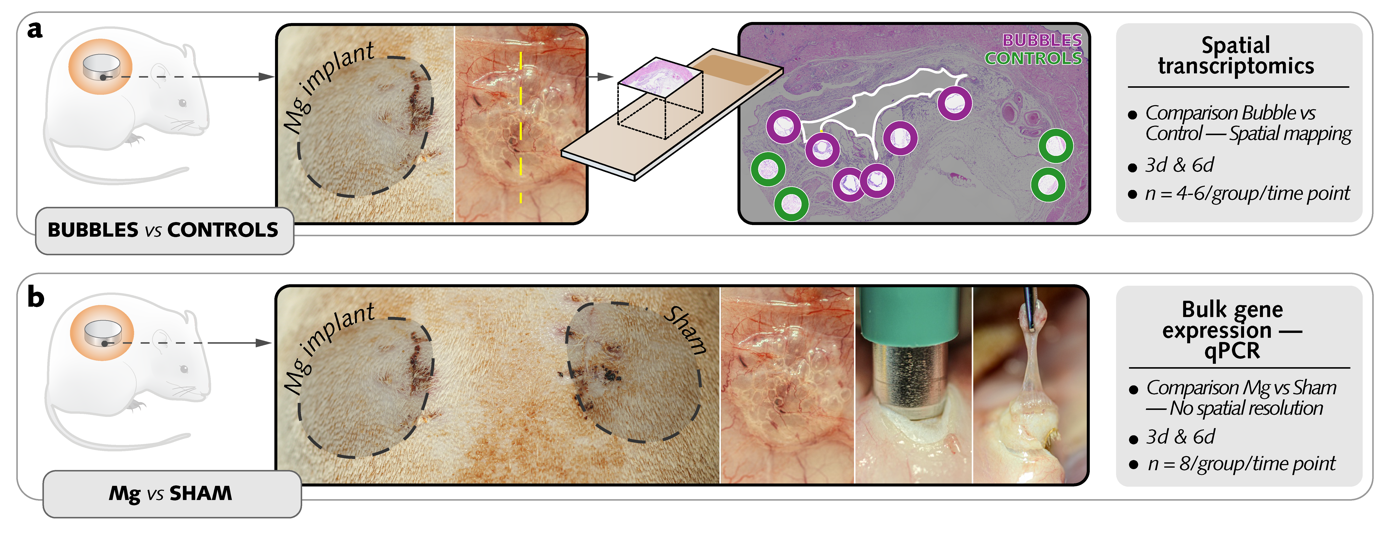
***

***Figure S11:* Spatial transcriptomics and bulk gene expression studies**

**a,** Spatial transcriptomic analysis (top panel) compared Bubble versus Control regions to identify localized molecular changes around bubbles at 3 and 6 d. Regions of interest (ROIs) were identified and processed to generate spatially resolved gene expression profiles. **b,** Selected genes with high differential expression from spatial transcriptomics were further analyzed using bulk gene expression studies via quantitative PCR (qPCR), comparing magnesium implants with sham implants, to determine whether the molecular fingerprint around bubbles was also relevant to the broader tissue regions surrounding the implant.


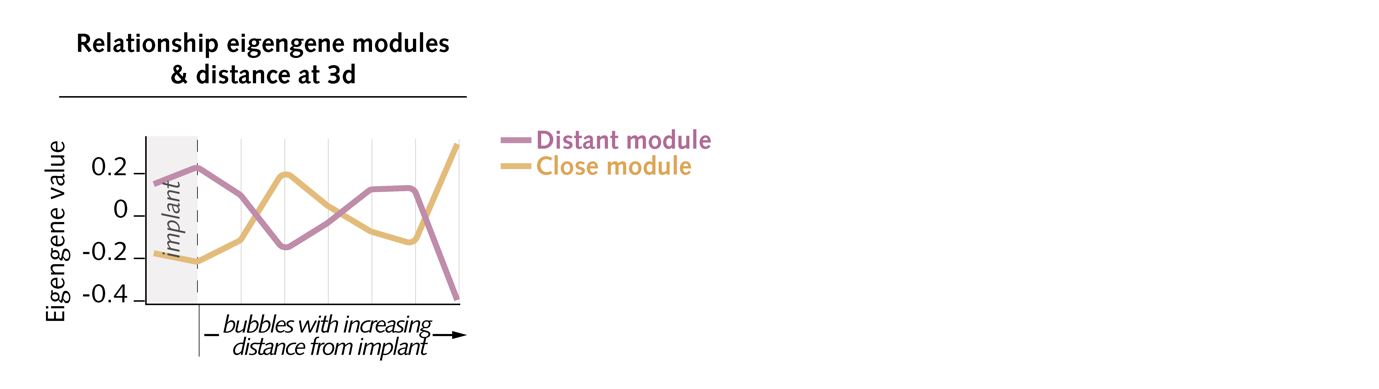
***Figure S12:* Relationships between eigengene modules and the bubble–implant distance at 3 d**

Relationship between eigengene values and distance from the implant at 3 d, without clear trends for the "Distant" (immune-related) and "Close" (matrix-related) modules.

***
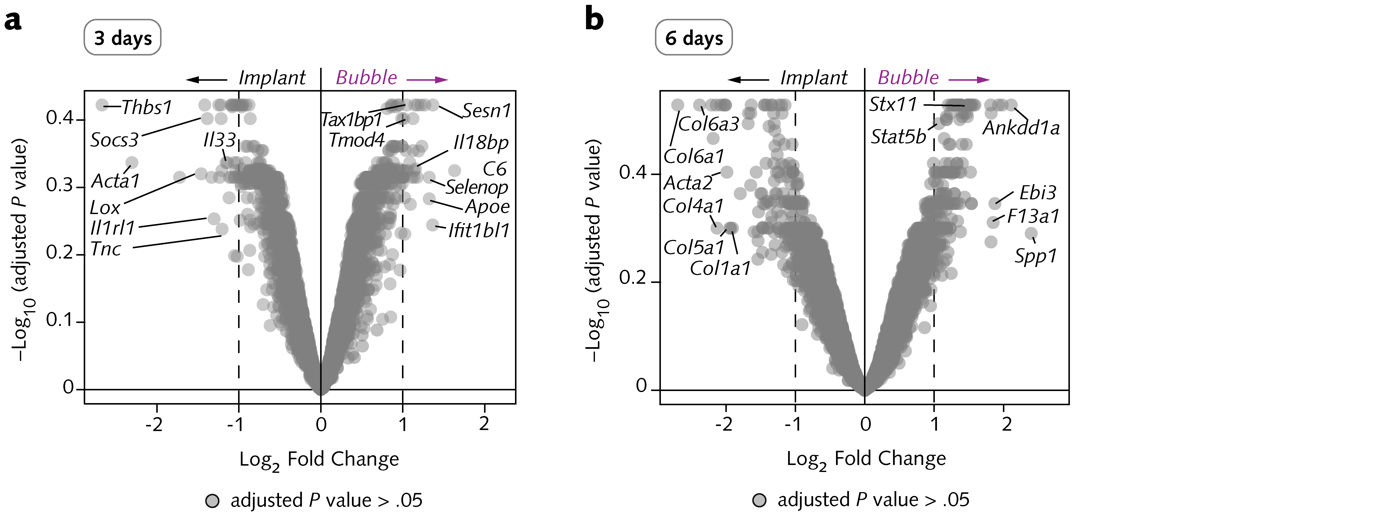
***

***Figure S13:* Differential gene analysis between the Bubble and Implant groups at 3 days and 6 days**

**a,** Volcano plots depicting the fold changes and significance of differentially expressed genes in the Implant (left) and Bubble (right) groups at 3 d (No gene expression had a significant difference between Bubble and Implant groups). **b,** Volcano plots depicting the fold changes and significance of DEGs in the Implant (left) and Bubble (right) groups at 6 d. Differential expression analysis was conducted via linear models (No gene expression had a significant difference between Bubble and Implant groups).

Statistical significance: *P* < 0.05 was adjusted via the Benjamini‒Hochberg procedure.

***Table S1: Primary antibodies and dilutions for immunohistochemistry and immunofluorescence***

| **Target Protein** | **Host / Isotype** | **Dilution** | **Supplier / Cat. No.** | **Clone No.** | **RRID** |
| --- | --- | --- | --- | --- | --- |
| Inducible nitric oxide synthase (iNos) | Rabbit polyclonal IgG | 1:100 | Thermo Fisher, PA1036 | / | AB_325773 |
| Mannose receptor C-type 1 (Mrc1) | Rabbit polyclonal IgG | 1:30 | Thermo Fisher, PA5101657 | / | AB_2851091 |
| Cluster of differentiation 68 (Cd68) | Rabbit polyclonal IgG | 1:3,000 | Thermo Fisher, PA581594 | / | AB_2788792 |
| Piezo type mechanosensitive ion channel component 1 (Piezo1) | Rabbit polyclonal IgG | 1:100 | Thermo Fisher, PA5-106296 | / | AB_2853973 |
| Collagen type VI (COL6) | Rabbit monoclonal IgG | 1:100 | Abcam, ab200429 | EPR17072 | / |

***Table S2: Bio‑Rad PrimePCR™ assays used for the qPCR analysis.***

| **Gene** | **Unique assay ID** | **Amplicon length (bp)** | **Efficiency (%)** | **Amplicon context sequence** |
| --- | --- | --- | --- | --- |
| *Myo1g* | qRnoCID0019265 | 119 | 101 | GGCCCATACAATGGCAGTTCCTGGTAGGGGTTCACAGATACCAGCACCTCGCCAATGTAGGTATAGATACGGCCCTTCTCAAACCTGAGCTGTAGGTTCTTCATAAACTCCTCCATTGTTAGTTGGTCCAAAAGCACAAAGTCTGGTTT |
| *Spp1* | qRnoCED0009101 | 64 | 99 | TGTGTGCTGGCAGTGAAGGACTCATCAGATTCATCGGAATGGTGAGATTCGTCAGATTCATCCGAGTTCACAGAATCCTCGCTCTCTGCATGGT |
| *Irf7* | qRnoCED0005624 | 77 | 99 | CCTTCTTTCAAGGGACTCCACAAGGTCCACTAGAGATGACATATAGCCAAGGAATAAGCCTGAGCCAGGGCAGCAGTGGTTCTGAACTCTATTGGAAGTTGGGGTTC |
| *Cybb* | qRnoCID0004581 | 117 | 101 | AAGATGGTAGCTTGGATGATAGCACTTCACACGGCCATTCACACCATTGCACATCTGTTCAACGTGGAGTGGTGTGTGAATGCCAGAGTCGGGACTTCGGACCCATATTCAGTAGCACTCTCTAACATTGGAGACAAAGAAAATGAA |
| *Gapdh* | qRnoCID0057018 | 115 | 96 | TGATGGCAACAATGTCCACTTTGTCACAAGAGAAGGCAGCCCTGGTAACCAGGCGTCCGATACGGCCAAATCCGTTCACACCGACCTTCACCATCTTGTCTATGAGACGAGGCTGGCACTGCACAAGAAGATGCGGCTGTCTCTA |
| *Hprt1* | qRnoCED0057020 | 79 | 98 | TTCATGCAAAAGCTTTACTAAGTAGATGGCCACAGGACTAGAACGTCTGCTAGTTCTTTACTGGCCACATCAACAGGACTCTTGTAGATTCAACTTGCCGCTGTCTTTT |
| *Actb* | qRnoCID0056984 | 74 | 97 | CGTCCACCCGCGAGTACAACCTTCTTGCAGCTCCTCCGTCGCCGGTCCACACCCGCCACCAGTTCGCCATGGATGACGATATCGCTGCGCTCGTCGTCGACAAC |
